# Genetic Dynamics of Allotetraploid Cotton Species (*Gossypium* spp.) and Their Utilization in Breeding for Superior Fiber Quality

**DOI:** 10.3390/plants15182866

**Published:** 2026-09-19

**Authors:** Dilrabo K. Ernazarova, Madina D. Kholova, Doston Sh. Erjigitov, Feruza U. Rafieva, Abdulqahhor Kh. Toshpulatov, Barno B. Oripova, Asiya K. Safiullina, Sevara K. Arslanova, Durdona B. Sokiboeva, Gavkhar F. Mamatkulova, Ezozakhon F. Nematullaeva, John Z. Yu, Fakhriddin N. Kushanov

**Affiliations:** 1 Institute of Genetics and Plant Experimental Biology, Academy of Sciences of the Republic of Uzbekistan, Tashkent 111208, Uzbekistan; edilrabo64@gmail.com (D.K.E.); mxolova107@gmail.com (M.D.K.); dostonerjigitov68@gmail.com (D.S.E.); feruzarafiyeva25@gmail.com (F.U.R.); toshpolatovabduqahhor78@gmail.com (A.K.T.); barnooripova127@gmail.com (B.B.O.); asiyasafiullina0996@gmail.com (A.K.S.); arslanovasevara87@gmail.com (S.K.A.); durdonasoqiboyeva@gmail.com (D.B.S.); gavhar0411@gmail.com (G.F.M.); ezoza.fn@gmail.com (E.F.N.); 2 Department of Genetics, Institute of Biochemistry, Samarkand State University Named After Sharof Rashidov, Samarkand 140104, Uzbekistan; 3 Department of Histology and Medical Biology, Chirchik Branch of Tashkent State Medical Academy, Chirchik 111727, Uzbekistan; 4 Department of Histology and Medical Biology, Tashkent State Medical University, Tashkent 100109, Uzbekistan; 5 United States Department of Agriculture (USDA)-Agricultural Research Service (ARS), Southern Plains Agricultural Research Center, College Station, TX 77845, USA; 6 Department of Microbiology and Biotechnology, National University of Uzbekistan Named After Mirzo Ulugbek, Tashkent 100174, Uzbekistan

**Keywords:** *Gossypium*, allotetraploid, cytogenetics, phylogenetics, meiosis, transgressive segregation, interspecific hybridization, fiber quality

## Abstract

The restricted genetic diversity of current cotton varieties (*Gossypium hirsutum* L.) considerably limits breeding opportunities aimed at enhancing fiber quality and resistance to environmental factors. Wild types of allotetraploid cottons are valuable sources of genetic variation, and their breeding potential has not yet been fully utilized. In this study, the inheritance and variability of important agronomic traits were analyzed in F_1_ and F_2_ hybrid generations obtained from intra- and interspecific crosses involving *G. hirsutum*, *G. barbadense*, and *G. darwinii*. The phylogenetic relationships of allotetraploid species were analyzed using molecular-genetic and cytogenetic methods, and their breeding potential was evaluated. Cytogenetic analysis of the F_1_ hybrids revealed the occurrence of univalents at metaphase I of meiosis, while bivalent numbers reached 25.9 in some hybrid combinations. Based on molecular marker analysis, significant genetic differences and polymorphism were identified between var. *gambia* and var. *m. palmerii*. There were reciprocal translocations, shown as multivalents, during ecotypic differentiation in other cross combinations. Positive transgressive segregation for the enhancement of fiber length was identified in F_2_ populations. Notably, in certain hybrid combinations, fiber length extended to 40.9 mm. Furthermore, molecular analysis identified 113 unique alleles across the genotypes examined. These findings affirm that wild and exotic allotetraploid cotton forms constitute a significant source of genetic diversity for breeding programs.

## 1. Introduction

The genus of *Gossypium* L. (family *Malvaceae*, tribe *Gossypieae*) serves as an evolutionary model for studying processes of polyploidization, genome evolution, and phenotypic diversification in the plant kingdom [1,2,3]. Distributed mainly across arid and semi-arid, tropical, and subtropical regions worldwide, this genus encompasses more than 50 species [4,5]. Taxonomically and cytogenetically, they are classified into eight diploid groups (A-G and K n = x = 13) and at least seven independent allotetraploid lineages (AD_1_–AD_7_ n = 2x = 26) [6,7,8]. According to molecular genetic data, allotetraploid cottons originated in the Pleistocene (1–2 million years ago) through interspecific hybridization between an Old-World A-genome diploid ancestor (close to *Gossypium herbaceum* L. or *Gossypium arboretum* L.) and a New World D-genome diploid ancestor (close to *Gossypium raimondii* L.) and subsequent chromosome doubling [9,10]. Recent studies confirm that the most extensively grown tetraploid cotton (*Gossypium hirsutum*) was domesticated in the Yucatán Peninsula (México) [11]. Furthermore, phylogenetic analyses have clarified the evolutionary relationships among tetraploid cotton species and highlighted their genetic potential for broadening the breeding pool. Comparative genomic studies revealed significant divergence patterns and adaptive traits valuable for modern cotton improvement [12].

Despite the vast morphological and ecological diversity present in the wild and exotic gene pool, the modern global cotton industry relies on an extremely narrow genetic base [13]. Over 90% of the world’s cotton fiber production comes from cultivars belonging solely to one species, *G. hirsutum* (upland cotton), while the remainder is largely attributed to *Gossypium barbadense* (Pima or extra-long staple cotton) [14]. Centuries of unidirectional artificial selection targeting key agronomic traits such as yield, earliness, and fiber properties have resulted in an intense genetic ‘bottleneck’ in cotton [15]. Consequently, genetic variation among cultivated varieties has dramatically decreased, making them highly susceptible to extreme drought and emerging diseases. To overcome this genetic plateau, introgressing new alleles from wild, exotic, and tropical semi-wild allotetraploid forms into the genomes of cotton cultivars is among the most pressing tasks today. Wild cotton germplasms such as *Gossypium darwinii* Watt., and wild subspecies of *G. hirsutum* (ssp. *mexicanum*, ssp. *punctatum*) stand out for their robust physiological immunity, deep root systems, and unique fiber quality [4]. These wild relatives represent an important source of novel alleles for improving drought tolerance, pest resistance, and fiber quality in cultivated cotton. Recent hybridization studies demonstrated their high potential for broadening the genetic base and enhancing adaptive traits in modern breeding programs [16]. However, the main obstacle to systematic use of these wild genomes in conventional and marker-assisted selection programs is the limited characterization of their subspecific taxonomy and the mechanisms of chromosomal divergence.

The morphogeographic classification system founded by Mauer (1954) and later refined by Fryxell (1992) laid the groundwork for understanding cotton evolution [1,6]. Yet, modern molecular phylogenies and high-resolution cytogenetic studies now necessitate a revision of traditional species boundaries [8]. For example, molecular confirmation of *G. ekmanianum*, an endemic of the Dominican Republic as a distinct AD_6_ lineage, indicates the existence of active cryptic speciation processes within the allotetraploid complex [14]. During prolonged periods of geographic and ecological isolation, wild populations accumulate chromosomal rearrangements, such as reciprocal translocations and inversions, which reduce pollen fertility and lead to partial sterility in hybrids. The development of synthetic allotetraploids between diploid cotton species has further expanded opportunities for introducing novel allelic combinations into cultivated cotton, significantly enhancing the genetic diversity available for breeding and facilitating the transfer of economically important traits [17].

When these primitive plant samples are crossed with cultivated ssp. *euhirsutum* varieties, structural differences manifest in the chromosomes of F_1_ hybrids during microsporogenesis as univalents, bivalents, and quadrivalents, resulting in partial pollen sterility. However, if these cytogenetic and reproductive barriers are systematically identified and eliminated, most genetic variation can be unlocked in subsequent segregating populations. This often leads to positive transgressive segregation for quantitative traits such as fiber length and boll weight [18,19]. Recent gene pyramid strategies and meta-QTL analyses have shown that the combination of favorable loci can significantly improve fiber quality and agronomic performance in cotton breeding populations [20,21].

To address these fundamental challenges, this study evaluated the phylogenetic relationships, cytogenetic stability, and breeding potential of various wild and exotic allotetraploid cotton resources. By observing chromosome conjugation at meiosis metaphase-I, analyzing molecular polymorphism using SSR markers, and monitoring inheritance of valuable agronomic traits in the segregating progenies, the biotechnological basis for exploiting wild germplasm was established. In addition, integrated morphological and molecular approaches have proven effective for assessing heritability and genetic variability in breeding populations, improving the efficiency of genotypic selection [22,23,24].

Previous studies have shown that fiber length is an important component of cotton fiber quality and has a high heritability in crosses between closely related forms and species [25,26].

However, the inheritance and variability of fiber length and other economically valuable traits in offspring obtained from crosses involving genetically diverse tetraploid forms have not yet been sufficiently studied [17]. Therefore, the purpose of this study was to determine the phylogenetic relationships and genetic diversity of the studied specimens, to evaluate cytogenetic compatibility and reproductive stability between them during intra- and interspecific hybridization, and to study the inheritance and variability of key economically valuable traits in F_1_ and F_2_ generations. The results obtained can serve as a genetic basis for expanding the genetic base of tetraploid cotton species and its effective use in improving fiber quality and economically important traits.

## 2. Results

### 2.1. Intraspecific and Interspecific Hybrids

To determine the phylogenetic relationships among the cotton samples, initial intraspecific and interspecific hybrids were obtained. For the wild-type crosses of *G. hirsutum* with each other and with cultivated medium-fiber varieties, the hybrid boll formation rate and, based on this, the degree of crossability and reproductive compatibility were analyzed.

When the wild and exotic subspecies (ssp. *mexicanum*, ssp. *punctatum*) of *G. hirsutum* were crossed with the cv. AN-Bayavut-2, they exhibited a high degree of genetic proximity. Out of 36 hybridizations between var. *m. palmerii* and cv. AN-Bayavut-2, 24 hybrid bolls were obtained, and the hybridization coefficient was 66.8%. In these hybrid bolls, the average full seed setting rate was 72.6%. Out of 30 crosses between AN-Bayavut-2 and ssp. *punctatum*, 23 hybrid bolls were obtained, with a hybridization rate of 76.7%, and the rate of full seed setting in hybrid bolls was 78.0%. The lowest result among the intraspecific hybridizations within *G. hirsutum* was observed in the combination of var. *gambia* × ssp. *punctatum*. Out of 31 crosses, 12 hybrid bolls were obtained, yielding a hybridization rate of 38.7%. The rate of full seed setting in hybrid bolls was 31.8% (Table 1).

In addition, the hybridization rate was analyzed in the combinations ssp. *punctatum* × var. *m. galante* and the reciprocal var. *m. galante* × ssp. *punctatum*, amounting to 51.4% and 62.5%, respectively. In these combinations, the full seed setting rate in hybrid bolls was 72.7% and 74.6%, respectively. A relatively low value was observed in the var. *gambia* × ssp. *purpurascens* combination, where out of 30 crosses, 11 hybrid bolls were obtained, the hybridization rate amounted to 36.7%, and the full seed setting rate in hybrid bolls was 41.7%. (Table 1).

Hybrids between intraspecific varieties of *G. hirsutum* and *G. barbadense* had moderate observed hybridization rates (within the range of 45.0–56.0%). No markedly distinct rates were observed for the development of fully matured seeds in the hybrid bolls, with all indexes identified as being in the range of 38.8–51.1% (Table S1).

Hybridization rates in interspecific crosses between *G. hirsutum* and *G. darwinii* were somewhat lower. In the cross between *G. darwinii*. and var. *m. palmerii*, eight hybrid bolls were obtained from 21 hybridizations, corresponding to a hybridization rate of 38.1%, while the proportion of fully developed seeds in the hybrid bolls was 45.0%. Similarly low results were seen in the cross of *G. darwinii* × var. *hopi*. Here, five hybrid bolls were obtained from 17 hybridizations, with a hybridization rate of 29.4% and the rate of fully developed seeds in hybrid bolls was 43.0% (Table S1). Hybrids from interspecific crosses between various forms of *G. hirsutum* and the wild species *G. darwinii* produced four hybrid bolls out of the 16 hybridizations (25.0% of the hybridization rate) of the *G. darwinii* × var. *el-salvador* combination, while the average rate of fully developed seeds in hybrid bolls was 44.2%. In the reciprocal var. *el-salvador* × *G. darwinii* cross, the hybridization rate averaged 31.8%, and the average rate of fully developed seeds in hybrid bolls was 42.2%.

Interspecific crosses between accessions of *G. barbadense* and *G. darwinii* generally demonstrated higher compatibility compared to the other two groups involving *G. hirsutum*. A relatively low result was observed only in the cross between *G. darwinii* and f. *parnat*, where the hybridization rate was 33.3%, although the rate of fully developed seeds in hybrid bolls was notably high at 90.4%. In its reciprocal combination, the hybridization rate reached 60.0%, with an average of 83.5% fully developed seeds in hybrid bolls. Similarly, in the *G. darwinii* × f. *brasiliense* and its reciprocal (f. *brasiliense* × *G. darwinii*) combinations, the crossability indices were 70.0% and 63.2% respectively, while the percentage of fully developed seeds in hybrid bolls was 82.3% and 73.5%, respectively. (Table S1).

### 2.2. Inheritance and Variability of Boll Weight in Intraspecific and Interspecific F_1_ and F_2_ Hybrid Progenies

**Figure 1:**
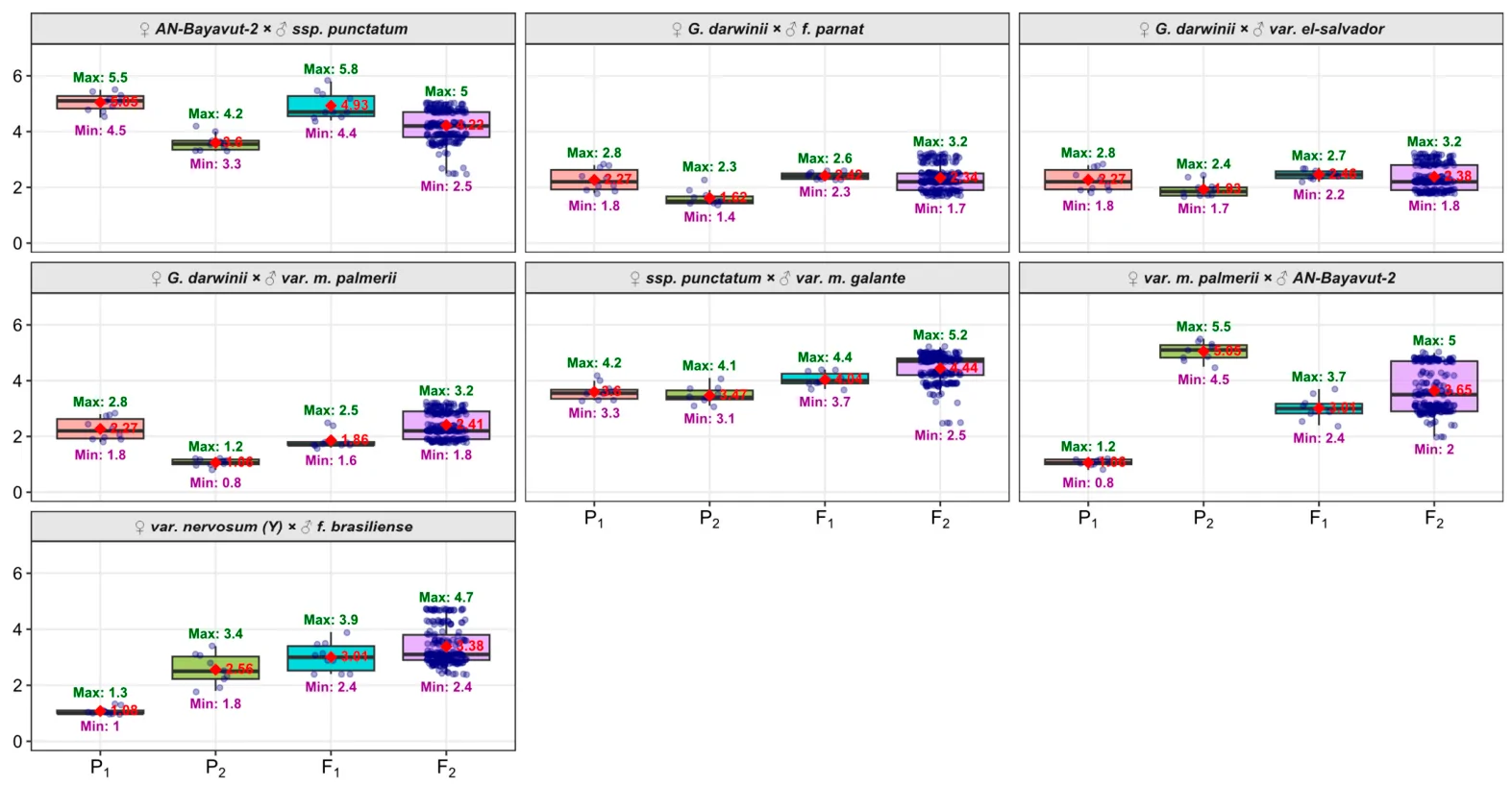
Variation and distribution of boll weight (g) across parental genotypes (P_1_, P_2_) and hybrid populations (F_1_, F_2_) in seven cotton cross combinations. Boxplots show the interquartile range (IQR, 25–75th percentiles) with the median indicated by the horizontal line inside the box. Red diamonds with numerical labels represent population means (M). Green labels indicate maximum values, purple labels indicate minimum values, and individual dots represent single plant observations.

Inheritance and variability of fiber weight per boll were analyzed in parental forms as well as in F_1_ and F_2_ hybrid progenies. The results of the analysis showed significant differences among parental samples for this trait, with fiber weight per boll recorded in the range of 0.8 g to 5.5 g (Figure 1).

In ssp. *punctatum*, the average fiber weight per boll was 3.30 ± 0.14 g, and in ssp. *paniculatum* (R) it was 2.50 ± 0.14 g. The highest value was observed in cv. AN-Bayavut 2, with an average of 5.10 ± 0.10 g. For the remaining forms, this trait was relatively low, recorded in the range of 0.8–1.5 g (Table S2). In the cultivated tropical form f. *brasiliense* and in *G. darwinii*, the average fiber weight per boll was found to be 2.7 g and 2.3 g, respectively.

Among intraspecific F_1_ hybrid progenies, the crosses of var. *m. palmerii* × cv. AN-Bayavut-2 and cv. AN-Bayavut 2 × ssp. *punctatum* displayed higher boll weight values (3.0–4.9 g) compared to other hybrids, and it was determined that the trait was inherited in a partially dominant manner (hp = −0.05 and hp = 0.69). Among the intraspecific F_1_ hybrids, the inheritance of the trait exhibited the highest heterosis in the cross of var. *m. palmerii* × var. *nervosum* (V) (hp = 12.33). In interspecific F_1_ progenies of *G. darwinii* × var. *m. palmerii* (hp = 0.16) and f. *parnat* × *G. darwinii* (hp = −0.14), the cotton trait was intermediate, while in the remaining crosses, inheritance followed the heterosis type (Table S2).

In F_2_ generations, the boll weight ranged from 2.0 g to 5.4 g. Predominantly, plants with boll weight greater than 2 g were observed. Only in two interspecific F_2_ hybrids (*G. darwinii* × var. *m. palmerii* and *G. darwinii* × f. *parnat*) were small-boll plants (lint weight per boll less than 2 g) encountered. In these hybrids, the mean value of the trait was 2.3–2.4 g (Table 2).

In addition, large boll hybrids (with a fiber weight per boll above 5 g) were identified among the ssp. *punctatum* × var. *m. galante* Thus, it was established that the degree of heterosis in intraspecific hybrids depends on the genotype of the original parental forms. This indicates that there is a significant genetic difference between the parental forms used in the crosses.

### 2.3. Heritability and Variability of Fiber Length in Intra- and Interspecific F_1_ and F_2_ Hybrids

Applying statistical evaluation methods of heritability, variability, and genetic correlation provides objective information about the traits under study for the development of new cotton genotypes, expansion of variability, and increasing the intensity of breeding [27]. Studying the types of heritability and gene interaction is of great importance for accelerating the improvement of fiber quality in intra- and interspecific hybrids derived from crosses of elite cotton varieties. With respect to fiber length, uniformity index, and Micronaire index, specific (distinct) additive and epistatic effects have been observed. Therefore, the low heritability of qualitative traits indicates that selection aimed at improving these traits may begin with the F_2_ generation [28]. However, alternative scientific results demonstrate a high heritability of fiber quality traits in hybrids. In this study, the broad-sense heritability coefficient was found to be 0.73 for relative breaking strength, 0.78 for Micronaire, and 0.93 for uniformity index. In the selection process, attention should be paid to the fiber properties with high heritability, to successfully select genotypes with important economic traits. Another group of researchers also support a similar approach in assessing the heritability of fiber quality traits [29].

As reported in the literature [27] and presented in our study, we observed the phenomenon of heterosis and dominance of the long-fibered parental forms in the F_1_ hybrids, and a decrease in average fiber length was observed in the F_2_ generation. Moreover, the inheritance of fiber length differs between intra- and interspecific hybrids, varying depending on the genetic nature of the original forms and the cross combinations. The original forms belonging to the intraspecific diversity of *G*. *hirsutum* tend to have relatively shorter fiber length. Fiber length in wild and exotic forms varies from 22.2 to 28.5 mm, whereas cv. AN-Bayavut-2 is characterized by a relatively higher fiber length, 32.7 mm (Figure S1).

Fiber length in the studied forms ranged from 21.6 to 34.8 mm. Among the parental forms, the longest fiber length was observed in wild *G. darwinii*, reaching 38.4 mm. Forms belonging to *G. barbadense* (f. *parnat*, f. *i. nigeria*, f. *brasiliense*) had lower fiber length property (21.6–25.5 mm). At the next stage, the inheritance of fiber length in the F_1_ generation of intra- and interspecific hybrids was studied (Table S2). The table shows intermediate, dominant, and over-dominant inheritance, as well as a positive heterosis effect, were observed in intraspecific hybrid combinations.

In three intraspecific hybrid combinations, in particular: f. *palmerii* × var. *nervosum* (Y) (hp = 0.64), var. *florida* × var. *hopi* (hp = 0.44), and var. *m. palmerii* × cv. AN-Bayavut -2 (hp = 0.89), the fiber length trait displayed inheritance of an intermediate type, tending towards the longer-fibered parent. In the remaining seven intraspecific hybrids, however, the trait showed pronounced dominant inheritance, with the dominance coefficient amounting to hp = 1.26, 1.47, 1.86, 2.21, 3.45, 8.25, and 13.00, respectively (Table S2). In interspecific hybridization combinations, the trait exhibited intermediate, dominant, as well as, in most hybrids, over-dominant inheritance, with values exceeding the best parent. Predominantly, all interspecific hybrids obtained from *G. hirsutum* × *G. hirsutum* crosses displayed overdominance, except for two reciprocal hybrids: ssp. *punctatum* × var. *m. galante* (hp = 0.59) and var. *m. galante* × ssp. *punctatum* (hp = 0.69). In the combination f. *i. nigeria* × var. *nervosum* (Y), complete dominance of the best parent var. *nervosum* (Y) was observed for the trait (fiber length 25.8 mm, hp = 1.00) (Table S2).

Our results confirm that in the F_1_ plants, fiber length is predominantly controlled by dominant genes. This is followed by a decrease in fiber length in all F_2_ progeny (Table S4). The observed overdominance for fiber length is consistent with previous QTL studies showing that overdominant and epistatic genetic effects can substantially contribute to heterosis for fiber quality traits in upland cotton [30,31].

In this study, the variation range of fiber lengths in F_2_ hybrid combinations was from 23.0 mm to 38.9 mm. For example, in the combination *G. darwinii* × var. *m. palmerii*, both short-fibered (24.0–26.9 mm) and long-fibered (36.0–38.9 mm) forms were observed to segregate. The mean value of this trait was 30.0 mm, with a coefficient of variation (COV) of 13.84% (Table S3). In the F_2_ progeny of *G. darwinii* × f. *parnat*, long-fibered forms (38.0–40.9 mm) accounted for 4.5% of the total studied plants (Table S4). The F_2_ progeny derived from the cross between var. *m. palmerii* × AN-Bayavut-2 was notable for its narrow range of fiber length variability (four classes). The majority (48.7%) of F_2_ progeny had fiber length falling within the average class (27.0–29.9 mm). The mean trait value was 29.0 mm, with a COV being 10.8%. Thus, progeny analysis showed that in the F_1_ hybrids obtained from intraspecific and interspecific crosses, the fiber length is mainly inherited as positive overdominance. In the F_2_ progeny, a decrease in fiber length was observed in all hybrid combinations (Table S4).

The results of this study show that it was possible to identify valuable long-fibered transgressive forms with a fiber length of 38.0–40.9 mm from the segregating populations in the following combinations: F_2_ *punctatum* × var. *m. galante* (3.92%), F_2_ *G. darwinii* × var. *m. palmerii* (5.5%), F_2_ *G. darwinii* × var. *el-salvador* (4.0%), and F_2_ *G. darwinii* × f. *parnat* (4.5%) (Table S4).

### 2.4. Inheritance and Variability of Fiber Yield in Intraspecific and Interspecific F_1_ and F_2_ Hybrids

Fiber yield is considered a polygenic trait, directly dependent on seed weight, fiber index, as well as the number, length, and weight of fibers. Accordingly, the correct selection of appropriate parental forms in hybridization [28] and their optimal compatibility in hybrid combinations serve as key factors. Strong segregation occurs for this trait in the F_2_ progenies. It has been established that the highest fiber yield parameters are observed when the parental forms are characterized by significant contrast in seed weight and fiber index.

In our study, fiber yield in parental forms ranged from 15.7% to 33.3%. Among the representatives of the species *G. hirsutum*, the lowest fiber yield was observed in the wild form var. *nervosum* (Y) (15.7%), and the highest in var. *gambia* (33.3%). The varieties of *G. hirsutum* demonstrated almost no difference in the studied trait and showed variation within a narrow range from 27.4% to 31.0% (Figure S2, Table S3).

Analysis of fiber yield showed that the intra- and interspecific F_1_ hybrids are characterized by inheritance according to both negative and positive heterosis types, and that hybrid plants exhibiting partial, complete, and overdominance also occur.

In the studied hybrid combinations of the F_2_ progeny, a decrease in the mean value of fiber yield was observed, from 26.6% to 29.5%. In the F_2_ progeny, the frequency of segregation of plants with higher fiber yield relative to the parental forms (29.0–34.9%) ranged from approximately 1.5% to 46.1% (Table S5).

For the fiber yield, the largest range of variability was observed in the F_2_ cv. AN-Bayavut 2 × ssp. *punctatum* combination (25.0–34.9%). In the 4th and 5th classes of this combination, the proportion of plants with a fiber yield above 30.0% reached 31.8% (Table S4).

Particularly in the reciprocal hybrid var. *m. galante* × ssp. *punctatum*, the average fiber yield was 28.0 ± 0.06%, with a COV of 7.83%. In this combination, the frequency of plants with fiber yield of 31.0–32.9% was 16.7%, while in the reciprocal combination (F_2_ ssp. *punctatum* × var. *m. galante*), it was much lower (2.0%). This result indicates that the inheritance of the fiber yield trait may be influenced to a certain extent by cytoplasmic genes or the direction of genotypes chosen as parental forms.

The next noteworthy result in discriminating forms with high fiber yield was observed in interspecific hybrids involving *G. hirsutum*. In all hybrid combinations involving *G. darwinii* (F_2_ *G. darwinii* × var. *m. palmerii*, F_2_ *G. darwinii* × ssp. *purpurascens*, and F_2_ *G. darwinii* × f. *parnat*), the range of fiber yield variability was very narrow, with most plants (almost more than 90%) falling into low-value classes (25.0–28.9%). In these combinations, the average value ranged from 26.6% to 27.1%, with the frequency of forms displaying high fiber yield (31.0–32.9%) at a minimal level (from 1.5% to 3.5%). The COV demonstrated stability in the range from 5.10% to 6.07%. Similarly, narrow variability ranges were also observed in hybrids obtained from representatives of *G. barbadense*. For example, in the hybrid F_2_ f. *brasiliense* × var. *nervosum* (Y), not a single plant with a fiber yield above 31.0% was identified, and 89.0% of the population was in the range of 25.0–28.9%. Overall, in the F_2_ generation, the COV for fiber yield was low to moderate in all combinations (from 4.8% to 7.83%). This again confirms that the fiber yield is of polygenic nature and its inheritance is complex, manifesting in close association with other traits such as seed weight and fiber index. The results show that only combinations involving the An-Bayavut-2 cultivar and the var. *m. galante* form of *G. hirsutum* had the possibility to isolate valuable families and breeding lines with high fiber yield ranging from 31.0% to 34.9% (Table S5).

### 2.5. Inheritance and Variability of 1000-Seed Weight Trait in Intraspecific and Interspecific F_1_ and F_2_ Hybrid Generations

The 1000-seed weight was obtained to determine the sowing rate and to assess field emergence (Figure S3).

Inheritance and variability of the 1000-seed weight were evaluated in parent forms as well as F_1_ and F_2_ progenies. Among parent samples, the 1000-seed weight ranged from 50.5 g to 119.0 g. The lowest values were observed in var. *nervosum* (Y) (58.1 g), var. *el-salvador* (50.5 g) and var. *florida* (53.2 g). In contrast, cv. AN-Bayavut 2, the wild species *G. darwinii* and several exotic accessions var. *gambia*, ssp. *punctatum*, var. *m. galante*, f. *i. nigeria*, f. *brasiliense* exhibited 1000-seed weight values above 100 g (112.6–119.0 g) (Table S3).

In all intraspecific (*G. hirsutum* × *G. hirsutum*) F_1_ hybrids, the 1000-seed weight exhibited overdominance and heterosis of inheritance (hp = 1.74; 4.79; 4.96). Only in var. *m. palmerii* × AN-Bayavut 2 was the trait in an intermediate state (hp = 0.02), while in AN-Bayaut 2 × ssp. *punctatum* (hp = −2.05), ssp. *punctatum* × var. *m. galante* and their reciprocal hybrids, negative overdominance (hp = −1.23; −1.70) was recorded. In the remaining interspecific hybrids, the inheritance of the 1000-seed weight ranged from partial to complete dominance and overdominance. (Table S3).

The variability range of 1000-seed weight was also obtained in the F_2_ generation, as with other traits. This range spanned from 75.0 g to 104.9 g and was divided into five classes. Among the F_2_ hybrids, the hybrid of var. *m. palmerii* × AN-Bayavut 2 stood out for having the widest (complete) variability range in 1000-seed weight. In the remaining intra- and interspecific hybrid families, the seeds were distributed across the five classes (Table S6).

### 2.6. Cytogenetic Analysis of Chromosome Conjugation, Meiotic Stability, and Pollen Viability in F_1_ Cotton Hybrids

Analysis of chromosome conjugation at metaphase I showed a generally stable course of meiosis in selected hybrid combinations. The number of bivalents in the studied combinations ranged from 25.5 to 25.9, indicating almost complete conjugation of chromosomes (Table S7).

**Figure 2:**
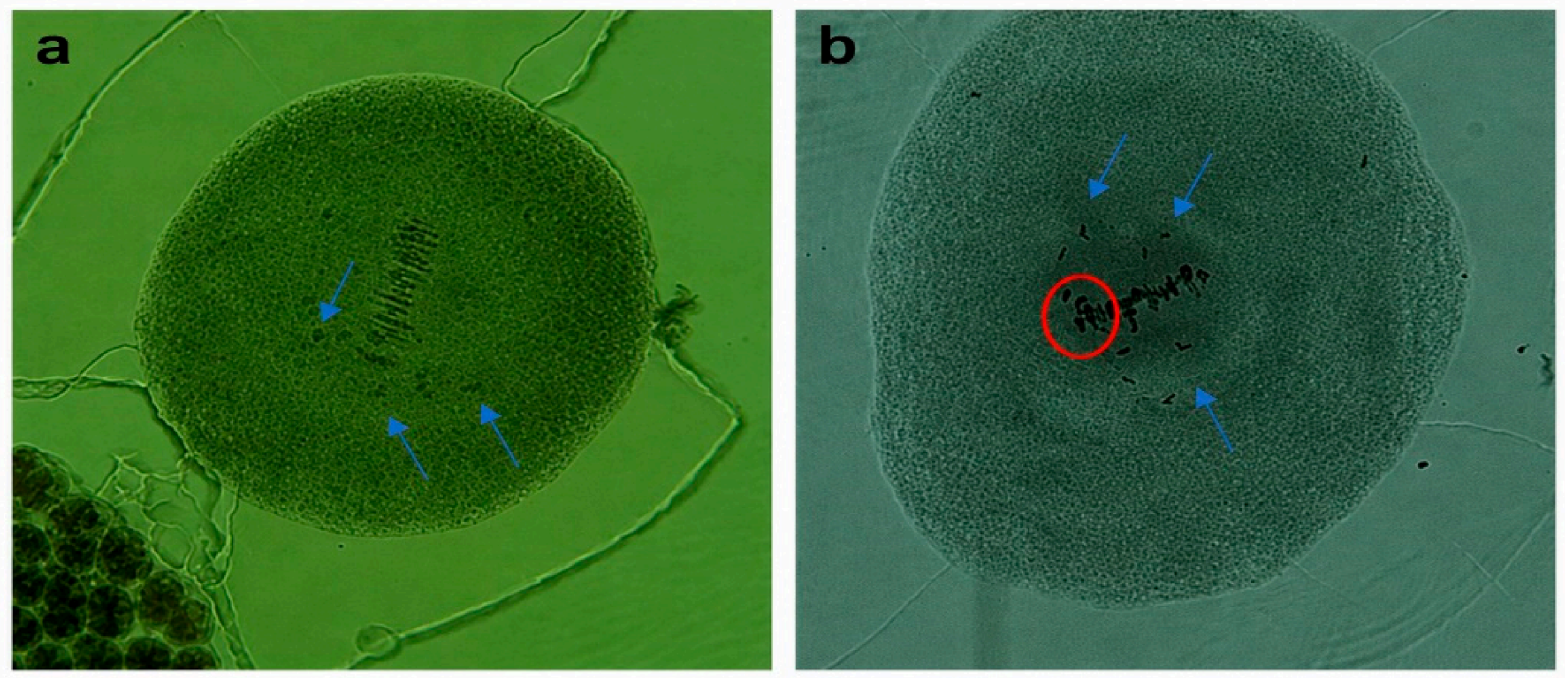
Chromosome conjugation at metaphase I in F_1_ hybrids of cotton: (**a**) var. *m. palmerii* × An-Bayavut-2; (**b**) f. *brasiliense* × *G. darwinii*. Univalents are marked with blue arrow, and quadrivalents are marked with red circle.

Among the hybrids studied, the combination var. *m. palmerii* × cv. AN-Bayaut 2 had the highest number of bivalents (25.8 ± 0.2) (Figure 2a) and the lowest number of univalents (0.4 ± 0.1). The combination cv. AN-Bayaut 2 × ssp. *punctatum* also had a high number of bivalents (25.5 ± 0.3), and almost complete conjugation of chromosomes was observed in this hybrid. The small number of univalents observed may be explained by the partial imperfection of conjugation characteristic of intergenomic hybrids and does not significantly affect the overall stability of the meiotic process (Table S7).

f. *ishan nigeria* × var. *hopi*, var. *nervosum* (Y) × f. *brasiliense*, *G. darwinii* × var. *el-salvador* and the reciprocal combination also showed that the number of bivalents was in the range of 25.58–25.92, respectively, and the number of univalents was very low (0.15–0.42) (Table S7). This result indicates a high degree of homology between the parental genomes of these hybrids.

In *G. darwinii* × f. *brasiliense* and its reciprocal combinations, no univalents were observed, and the number of bivalents was 25.70 ± 0.12 and 25.80 ± 0.10 (Figure 2b), respectively. Although a small number of quadrivalents (0.10–0.13) was noted in some cells, their low frequency did not affect the overall stability of the chromosome conjugation process. In general, the predominance of bivalents and the low occurrence of multivalents in all studied hybrid combinations indicated a relatively stable course of the meiosis and the promising selection prospects of these hybrids.

The results from the analysis of sporades showed that the meiotic process was relatively stable in the studied hybrid combinations. The number of analyzed sporades varied from 823 to 1484, depending on the hybrid combinations, and in most combinations the proportion of normal dyads, triads and tetrads was high.

Among the hybrids studied, the highest meiotic index in the combinations var. *m. palmerii* × AN-Bayavut-2 and ssp. AN-Bayavut-2 × ssp. *punctatum* were 90.16 ± 0.89% and 91.21 ± 0.78%, respectively. The absence of micronuclear tetrads and anomalous sporades in these combinations indicates that the meiotic process in these hybrids was highly stable (Table S8).

**Figure 3:**
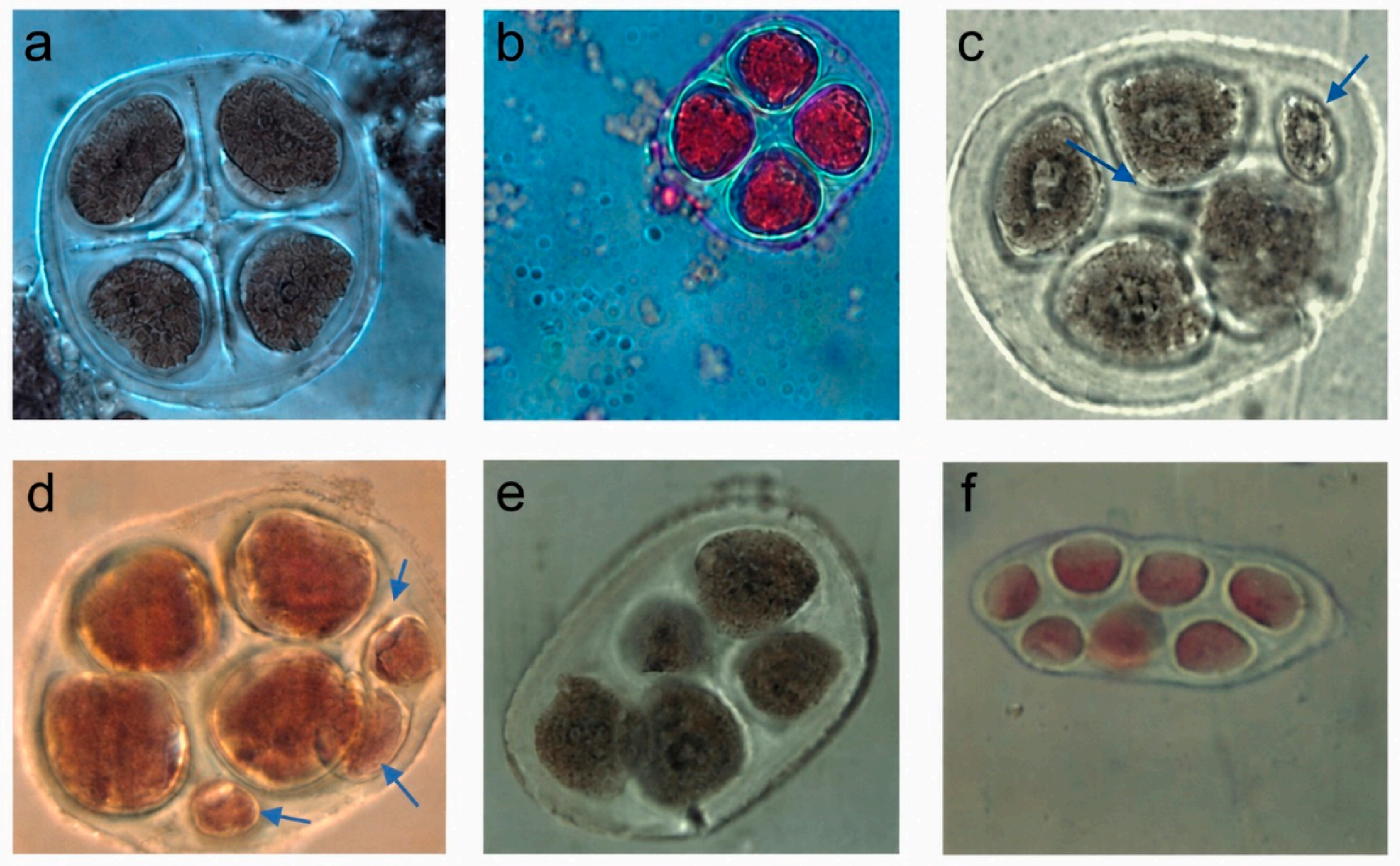
Normal and abnormal spores observed during microsporogenesis in F_1_ hybrids (in the case of the hybrid f. *i. nigeria* × var. *hopi*): (**a**,**b**) normal tetrads; (**c**,**d**) tetrads with micronuclei; (**e**) pentad; (**f**) heptad (micronuclei are indicated by arrows).

The combinations of f. *i. nigeria* × var. *hopi* and var. *nervosum* (Y) × f. *brasiliense* had a relatively low meiotic index, amounting to 84.10 ± 0.95% and 84.20 ± 0.98%, respectively. In these combinations, the proportion of micronuclear tetrads was 1.89–1.93, and the proportion of abnormal sporads was 3.29–3.30%. This indicates that some disturbances in the segregation of chromosomes occurred during meiosis (Table S8, Figure 3).

The meiotic index in the combination of *G. darwinii* × var. *el-salvador* and its reciprocal was 87.00 ± 1.00% and 86.48 ± 1.00%, respectively. In these combinations, the proportion of micronuclear tetrads was 1.95–2.15%, and anomalous sporades was 2.92–3.10%. At the same time, it was found that in the combination *G. darwinii* × f. *brasiliense* and its reciprocal, although micronuclear tetrads were not observed, the proportion of anomalous sporades was relatively high (4.13–4.18%) (Table S8).

In general, the meiotic index was in the range of 84.10–91.21% in the studied hybrid combinations. The predominance of normal spores and the small number of anomalous spores showed that the meiotic process was relatively stable and that these hybrids are promising for use in further selection work.

The results from the analysis of pollen fertility showed that gamete viability was relatively high in the studied interspecific hybrid combinations. For analysis, four flowers were taken from each combination, and the total number of pollen grains evaluated ranged from 1859 to 2714 (Table S9).

Among the hybrids studied, the highest pollen fertility index (94.12 ± 0.54%) was recorded in the combination of AN-Bayavut 2 × ssp. *punctatum*. Also, the fertility level was high in the combination of var. *m. palmerii* × AN-Bayavut-2, which amounted to 90.82 ± 0.57%. These results indicate that the meiotic process in these hybrids was stable and a high proportion of functional gametes was formed (Table S9).

The pollen fertility of the combinations f. *i. nigeria* × var. *hopi* and var. *nervosum* (Y) × f. *brasiliense* was 82.42 ± 0.73% and 83.04 ± 0.77%, respectively. At the same time, the fertility level of *G. darwinii* × var. *el-salvador* and its reciprocal combinations was relatively low, equal to 81.66 ± 0.90% and 82.14 ± 0.88%, respectively (Table S9).

*G. darwinii* × f. *brasiliense* and its reciprocal combinations had pollen fertility of 84.05 ± 0.70% and 84.52 ± 0.71%, respectively. In general, the pollen fertility in the studied hybrid combinations ranged from 81.66 to 94.12% (Table S9), indicating that reproductive processes in interspecific hybrids were relatively stable and they could be effectively utilized in the breeding programs.

Results from cytogenetic analysis showed that in the interspecies F_1_ hybrids studied, chromosomes were highly conjugated, the meiotic process was relatively stable, and pollen grain fertility was preserved at a high level, indicating that these hybrids are a promising resource for cotton breeding.

### 2.7. Analysis of Genetic Polymorphism and In Silico Annotation of Hybrid Populations and T-Genotypes Using SSR and SNP Markers

Among the DNA markers tested to evaluate baseline genetic variation in the parental germplasm, 55.5% were polymorphic. Specifically, only 9 out of the 25 SSR markers were polymorphic, while 16 (64%) were monomorphic among the parents. In contrast, the gene-specific markers demonstrated a significantly higher polymorphism rate, with 16 out of 20 loci (80%) showing polymorphic patterns and only 4 (20%) remaining monomorphic.

Unique alleles were identified for each locus, with a total of 113 unique alleles being detected. The largest number of unique alleles was observed during amplification with the NAU3284 and BNL0840 primers. Based on the frequency of detected alleles, the polymorphism information content (PIC) index was calculated for each marker. The PIC values ranged from 0.37 (NAU3284) to 0.92 (BNL3255), with an average value of 0.54. Analysis of locus variability also revealed substantial differences in expected heterozygosity (He) values, which fluctuated within the range of 0.49–0.92, with an average value of He = 0.62 (Table S10).

To evaluate the genetic relationships among *Gossypium* species, a similarity dendrogram was constructed based on the analysis of 25 polymorphic loci, comprising 16 gene-oriented functional markers and 9 SSR markers. Based on the results from the UPGMA cluster analysis, the genotypes studied were divided into several major clusters clearly differentiated from each other based on genetic distance values (from 0.000 to 0.200) (Figure S3). The dendrogram separated all analyzed accessions into two major similarity groups:

The First Major Group: This group formed a cluster comprising *G. darwinii*, which is endemic to the Galápagos Islands, alongside the var. *nervosum* (V). The genetic distance between this cluster and the remaining germplasm was relatively high, exceeding the 0.162 threshold.

The Second Major Group: Representing the most diverse and structurally complex assemblage, this group comprised the majority of the *G. hirsutum* complex, including wild and exotic forms, together with representatives of *G. barbadense* representatives. Based on genetic similarity, this group was further divided into two distinct sub-clusters (Sub-cluster IIa and Sub-cluster IIb).

Sub-cluster IIa: This sub-cluster primarily clusters accessions that are genetically close to cultivated cotton forms. Notably, the elite Uzbek cultivar representative, An-Bayavut-2, was grouped together with ssp. *punctatum* and ssp. *purpurascens* (2). The genetic distance between them is only 0.044, confirming high genomic and allelic affinity. Furthermore, ssp. *punctatum* variants of diverse eco-geographical origins—such as var. *hopi*, var. *java*, and var. *gambia*—were distributed across different branches of the dendrogram, with their genetic distances ranging from 0.044 to 0.092 (Figure S4).

Sub-cluster IIb: One of the most notable patterns in the UPGMA dendrogram is the clustering observed around the 0.023 similarity level. Several wild forms of *G. hirsutum* are grouped within the same cluster as representatives of *G. barbadense*. For instance, f. *parnat* and f. *brasiliense* are positioned on the same cluster branch as var. *florida* and ssp. *purpurascens*. Additionally, the Nigerian accession f. *ishan*, var. *nervosum* (Y), and var. *m. palmeri* are grouped together at a genetic similarity level of 0.053 (Figure S3).

The UPGMA similarity analysis showed that subspecies represented by multiple accessions did not consistently form distinct clusters. Specifically, accessions of ssp. *punctatum* were distributed across several small clusters, indicating considerable genetic differentiation among the examined forms. Likewise, accessions of ssp. *purpurascens* were separated between different clusters, including the upper cluster and the lower cluster containing several *G. barbadense* forms. A similar pattern was observed for ssp. *mexicanum*: var. *nervosum* (Y) and var. *m. palmeri* clustered together, whereas var. *nervosum* (V) was clearly separated from the remaining accessions and formed a distinct group closely associated with *G. darwinii*. It should be noted that the observed clustering pattern relies strictly on the polymorphism data of the 25 specific loci evaluated in this study. Given the relatively limited number of molecular markers, this dendrogram may not fully represent the comprehensive, genome-wide phylogenetic landscape of the analyzed cotton accessions. If marker density is significantly increased in future studies—for instance, through high-density SNP genotyping or whole-genome sequencing (WGS) approaches—the positional arrangement of certain genotypes and the overall dendrogram topology could be partially altered, potentially refining the genetic clustering of specific semi-wild forms. Nevertheless, the combination of 16 gene-targeted and nine SSR markers utilized in this work provided initial genomic resolution to identify major patterns of genetic similarity and deliver a robust baseline genetic screening of the germplasm collection.

#### 2.7.1. Annotation and In Silico PCR of Candidate Genes in Marked Regions

To screen genomic regions located upstream and downstream of highly polymorphic microsatellite regions, a bioinformatics method to walk along a chromosome was employed based on the complete genome sequences of the tetraploid species *G. hirsutum*, *G. barbadense*, and *G. darwinii*.

Based on the results from the in silico PCR analysis, seven out of the nine microsatellite markers were virtually amplified in the genome. Table S11 shows the annotation of candidate genes located around the seven identified markers—NAU3478, NAU3284, NAU2951, NAU5212, BNL3255, BNL1604, and BNL1122—using flanking genomic regions of 50,000 nucleotides upstream and 50,000 nucleotides downstream from the marker region, totaling 100,000 base pairs (bp).

#### 2.7.2. Prediction of Candidate Genes (AUGUSTUS Analysis)

To preliminarily annotate potential candidate genes controlling economically valuable traits, the AUGUSTUS web-based software (version 3.5.0) was used. When the nucleotide sequences of the screened marker regions were comparatively analyzed against the model plant *Arabidopsis thaliana* genome database, a total of 72 potential candidate genes and their 77 transcript variants were identified (Table S12).

Analyses showed that some virtually amplified genes possess several transcript variants. For example, in the NAU3478 microsatellite region, eight candidate genes and their nine transcript variants were identified, with the total number of amino acid sequences amounting to 77, which were taken as a basis for further investigation.

#### 2.7.3. Identification of Protein Homology Using BLAST Analysis

To search for homology between the identified 77 amino acid sequences and known, previously annotated proteins in the database, analyses were performed on the NCBI international web resource using the BLASTP algorithm (version 2.17.0) for protein/protein pairwise alignment. As a result of the comparative analyses, several important functional proteins that may be involved in the formation of various agronomically valuable and morpho-biological traits of cotton plants were identified (Table S13).

Among the genes identified, the regions surrounding the NAU3478 and NAU2951 markers stood out as the most promising systems for future research. According to the functional genomics annotation, these loci were found to encode proline-rich receptor-like protein kinases (*PERK4*), glucose-6-phosphate translocator (*GPT2*), serine carboxypeptidases, and early nodulin-like proteins (*ENODL3*) [32].

Although the length of the extracellular domains of atPERKs differed significantly, the kinase phylogeny showed a high degree of sequence similarity, as expected. The extracellular domain of all AtPERKs includes Proline-rich regions of the Ser-Pro type (3-5), in which Ser-Pro (2-3) motifs predominate. The *PERK4* receptor a member of this family, connects the external receptor domain through the plasma membrane to the intracellular cytoplasmic kinase cascade in the external signal transduction system. This receptor type activates plant defense mechanisms in response to bacterial pathogens and triggers specific transcription factors [33].

*GPT2*: This translocator ensures plant adaptation to rapidly changing abiotic factors such as light, humidity, and CO2 concentration. Carbon exchange across the inner envelope of chloroplasts directly affects photosynthesis and growth rate. Under high CO_2_ conditions or when starch metabolism is disrupted, *GPT2* gene expression increases sharply, activating *glucose-6-phosphate* (*G6P/Pi*) exchange across the chloroplast membrane [34].

*ENODL3*: This gene belongs to the early nodulin-like protein (ENODL) subfamily of phytocyanins, and ENODL proteins have been found to be involved in the initial stages of nodule formation in plants, cell differentiation, modification of the cell wall, and the transport of various soluble compounds. The presence of these proteins in both leguminous and non-leguminous plants suggests that they may perform broader functions in plant development and interactions with the environment [18].

The present analysis indicated the previously unannotated SSR markers NAU3478 and NAU2951 are located in close proximity to candidate genes. Preliminary in silico annotation suggests these genes may be indirectly associated with molecular pathways involved in plant development.

## 3. Discussion

Wild cotton species and genetically diverse exotic germplasm represent a valuable reservoir of rare alleles that can help broaden the increasingly narrow genetic base of cultivated cotton [16,17]. In modern cotton breeding, prolonged directional selection, primarily for high yield and superior fiber quality, has substantially reduced the genetic diversity of cultivated *Gossypium* species [35]. Therefore, incorporating the rare alleles preserved in wild species and genetically diverse exotic germplasm into current breeding programs is of considerable scientific and practical importance [36]. The phenotypic and cytogenetic results obtained in this study, as well as the genotypic diversity identified using SSR markers, reaffirm the significant value of wild allotetraploid cotton resources for breeding research. These resources may provide valuable genetic material for broadening the genetic base of cultivated cotton and may contribute to the development of future breeding strategies aimed at improving economically important and fiber quality traits [16,17,35,36].

Genetic variability in quantitative traits is fundamental to improving the efficiency of plant breeding. The assessment of this variability commonly involves estimating broad- and narrow-sense heritability [22,37,38]. Narrow-sense heritability is more important in plant breeding, because it reflects the proportion of phenotypic variation attributable to additive genetic effects and therefore provides a better indication of the expected response to selection [37,38]. During sexual reproduction, parental genotypes contribute their haploid genomes to the progeny, whereas dominance and epistatic interactions are not transmitted as fixed combinations and may be disrupted and reassembled through recombination and segregation in subsequent generations.

Thus, a parental genotype whose superior performance is primarily determined by dominance and epistatic effects, rather than by additive genetic effects, is expected to have a limited ability to transmit its superior performance consistently to progeny in subsequent generations [25,30].

Estimating the additive component of genetic variance requires specialized experimental designs and is often labor-intensive. However, when dominance and epistatic effects are relatively small, broad- and narrow-sense heritability estimates may be similar [37,38].

Cotton fiber is a highly elongated single cell that originates from the epidermal cells of the seed coat [1,2]. Depending on fiber length, cotton is classified into short, medium, and long-staple categories, each associated with different technological and industrial applications [1,31].

Furthermore, differences in hybridization success rates and mature seed set among the various crossing combinations indicated that the degree of genetic compatibility among parental forms was not uniform. Specifically, crosses involving certain subspecies of *G. hirsutum* exhibited higher hybridization rates, whereas combinations involving *G. darwinii* generally exhibited lower reproductive compatibility [16,27,28].

These observations align with the results of our marker-based genetic similarity analysis. In the constructed similarity dendrogram, *G. darwinii* formed a distinct cluster separated from the cultivated allotetraploid forms, indicating a relatively low level of genetic similarity between these groups [12]. Several samples representing *G. hirsutum* subspecies were distributed across different clusters in the similarity dendrogram, indicating a genetically complex pattern of marker-based similarity. Such patterns may reflect the complex domestication history of *G. hirsutum*, historical and ongoing gene flow, and introgression among geographically and genetically differentiated populations, as well as inconsistencies between traditional morphological classifications and underlying genetic relationships [39,40]. In particular, the high level of genetic diversity within the *G. hirsutum* complex and the evolutionary relationships among various geographic populations may account for the scattered placement of these subtaxa in the similarity dendrogram [40,41].

The lower hybridization and seed-setting rates observed in crosses involving *G. darwinii* may reflect variation in reproductive compatibility between the parental forms. Such variation could potentially influence the efficiency of gene exchange between wild and cultivated genotypes. However, hybridization success is affected by multiple biological and experimental factors, and the present results do not provide direct evidence for specific genetic or structural barriers.

The large phenotypic variation and the manifestation of transgressive forms observed in certain hybrid combinations confirm the feasibility of introgressing valuable alleles present in wild forms into the cultivated cotton genome [16,35]. The high level of SSR polymorphism observed among the studied materials further reflects their substantial genetic diversity, particularly within the wild and genetically diverse donor forms [5,35]. One of the most prominent aspects of this research is the emergence of pronounced phenotypic variation and positive transgressive segregants in segregating generations, particularly for economically valuable traits such as fiber length and fiber turnout. The fact that the hybrids outperformed both parental forms may reflect the contribution of complementary gene action, epistatic interaction, and the expression of previously unexpressed or cryptic alleles inherited from the parental (wild) forms; however, these mechanisms were not studied in this research. [25,30]. The specific genetic mechanisms underlying this transgressive segregation could be explored more extensively in future studies.

In summary, the results obtained from this study support the hypothesis that wild allotetraploid forms of cotton represent invaluable breeding resources. Their genetic diversity, molecular phylogeny, and cytogenetic characteristics highlight their considerable potential for the development of novel cotton varieties with improved fiber quality and enhanced resilience to biotic and abiotic stresses. These findings may also facilitate the identification of effective donor genotypes and the development of breeding strategies, including marker-assisted selection (MAS) programs.

## 4. Materials and Methods

### 4.1. Plant Samples and Field Trials

Field selection and hybridization experiments were conducted on the experimental plots of the Institute of Genetics and Plant Experimental Biology, Academy of Sciences of the Republic of Uzbekistan, under standard open field agrotechnical management. The research objects were the following wild, exotic, and cultivated allopolyploid (2n = 4x = 52) species of the *Gossypium* genus, as well as the hybrid progeny obtained from their crosses. The full botanical names of studied samples, as well as the abbreviations used to designate them in the article text, figures, tables, and Supplementary Materials, are provided below (Table 3):

Photoperiod-sensitive tropical and wild cotton forms were cultivated under special short-day conditions (10 h of light, 14 h of darkness) to induce transition to the flowering phase.

### 4.2. Hybridization and Evaluation of Valuable Agronomic Traits

Hybridization was carried out reciprocally, and the percentage of fully developed seeds in the resulting hybrid bolls was statistically analyzed according to the OriginPro 2021 [42] method. The data obtained for each trait were statistically analyzed using the modern variance ANOVA [43] program. The morphological and biological characteristics of research samples and their hybrid combinations were described using a specialized classifier [44]. Fiber length was measured for each sample by determining the seed fiber length on special Mauer boards. For the F_1_ hybrids analyzed, the degree of dominance (hp) for valuable agronomic traits was calculated using the Griffing [37] formula as cited in the scientific works of G. M. Beil and R. E. Atkins:

$$hp=\frac{\left(F1-MP\right)}{P-MP}$$

where hp is the degree of dominance;

F_1_ is the first filial generation’s arithmetic mean trait value;

MP is the mid-parent arithmetical mean trait value;

P is the arithmetical mean trait value of the best parent.

The inheritance of traits in F_1_ hybrids was assessed as follows:

(1) no dominance (hp = 0);
(2) partial dominance (0 < hp < 1);
(3) complete dominance (hp = 1);
(4) overdominance (hp > 1).

Broad-sense heritability (*H*^2^) of the traits in the F_2_ population was estimated according to the method of Warner (1952) [38]. Broad-sense heritability, defined as the proportion of total phenotypic variance attributable to genetic variance, was calculated as:

$$H^{2}=\;\frac{\sigma_{F_{2}\;}^{2}{-\;\sigma }_{E}^{2}}{\sigma_{F_{2}\;}^{2}}$$

where $\sigma_{F_{2}\;}^{2}$ is the phenotypic variance of the F_2_ population and $\sigma_{E\;}^{2}$ is the environmental variance.

The environmental variance was estimated from the mean variances of the two parental lines and the F_1_ generation as:

$$\sigma_{E}^{2}\approx \;\frac{\sigma_{P_{1}\;}^{2}+\sigma_{P_{2}\;}^{2}+\sigma_{F_{1}\;}^{2}}{3}$$

The estimated *H*^2^ value reflects the proportion of phenotypic variation in the segregating F_2_ population that is attributable to genetic effects and provides an estimate of the potential effectiveness of selection for the traits under investigation.

Only hybrid combinations that produced sufficient F_2_ progeny and exhibited high breeding potential in the F_1_ generation were selected for detailed evaluation in the F_2_ generation. These combinations involved genetically divergent parental forms and were considered the most suitable material for analyzing the segregation of economically important traits and identifying transgressive recombinants.

### 4.3. Cytogenetic Analyses and Meiosis Monitoring

Cytogenetic analyses are technically demanding and require the collection of flower buds at precisely defined developmental stages. In addition, differences in the reproductive development rate among the genotypes made it impractical to analyze all F_1_ hybrid combinations simultaneously. Therefore, two hybrid combinations were selected from each intra- and interspecific crossing group for comparative evaluation of chromosome pairing at metaphase I, sporad formation, and pollen fertility.

Cytogenetic analyses were performed according to the methods of Z.P. Pausheva [45] and R.P. Barykina [46]. To study chromosome conjugation during microsporogenesis, flower buds measuring 2–4 mm were collected from plants between 07:00 and 09:00 h. The buds were immediately fixed in freshly prepared Carnoy’s fixative (ethanol:acetic acid, 3:1, *v*/*v*) for 24 h, transferred to 70% ethanol for long-term storage, and kept at 4 °C. Chromosome configurations at metaphase I (MI) were examined in temporary preparations stained with 1% acetocarmine. For each hybrid combination, 50–100 pollen mother cells were analyzed under a light microscope, and the frequencies of univalents (I), bivalents (II), and quadrivalents (IV) were recorded. The meiotic index was calculated as the percentage of normal tetrads relative to the total number of sporads.

### 4.4. Molecular-Genetic and Bioinformatic Analyses

#### 4.4.1. Genomic DNA Extraction

Young leaves collected from cotton plant samples were frozen in liquid nitrogen and ground into fine powder. Genomic DNA was extracted from the leaf tissue using a slightly modified CTAB (cetyltrimethylammonium bromide) method according to Paterson et al. (1993) [47]. The concentration and purity of the isolated DNA were assessed using a NanoDrop Eight spectrophotometer (Thermo Fisher Scientific, Waltham, MA, USA) by measuring the absorbance at 260/280 nm. In addition, DNA quality was verified by gel electrophoresis and analyzed using Quantity One software (version 4.6.3, Bio-Rad, Hercules, CA, USA) through comparison with λ phage DNA of known concentration. All DNA samples were diluted to a working concentration of 25 ng/μL and stored at −20 °C until further molecular analyses.

#### 4.4.2. PCR Screening and Genotyping

A total of 45 DNA markers were utilized in this study. Among these, 25 SSR markers were retrieved from the CottonGen database, while the remaining 20 were gene-targeted markers specifically developed for identifying economically important agronomic traits [48]. The 10 μL PCR mixture was prepared with the following composition: 2 μL 5X ScreenMix (Evrogen, Moscow, Russia; Cat. №: PK041L), 1 μL forward and reverse primers (10 μmol), 1 μL (25 ng/μL) working concentration DNA, and 6 μL distilled water.

Polymerase chain reaction (PCR) was performed on a T100 Thermal cycler (Bio-Rad, Hercules, CA, USA). Amplification was carried out using a Hot-start program with the following time and temperature regime: initial denaturation at 94 °C for 20 s, denaturation at 94 °C for 20 s, primer annealing to the genome at 55–60 °C for 30 s, elongation at 72 °C for 50 s, followed by a final elongation at 72 °C for 5 min, conducted over a total of 42 cycles. Amplified PCR products were stored at 4 °C.

The amplified products were separated by 2.5% (*w*/*v*) agarose gel electrophoresis, and PCR amplicons were visualized based on ethidium bromide staining and photographed under ultraviolet light using the AlphaImager Gel documentation and analysis system (Bio-Rad, Cat. №: 1708265). Subsequently, fragment sizes were determined by comparative mobility with the 50 bp molecular weight marker (Bio-Rad, Cat No.: 170-8200).

To determine the genetic polymorphism of selected cotton genotypes within the framework of the study, a molecular-genetic analysis was conducted. The electropherogram results presented confirm the successful amplification of target fragments in 18 analyzed samples. The sizes of the obtained amplicons were determined by comparison with a 100 bp molecular weight marker (M).

### 4.5. Candidate Gene Identification

In silico PCR screening was performed using the genome sequence of the cotton species *G. hirsutum* [3] and microsatellite markers in the UniPro Ugene 1.21 [49] program. Here, the presence of the DNA markers in the cotton genome and their exact chromosomal positions were determined.

With the Augustus 3.5.0 bioinformatics program [50], probable genes and their transcript variants were identified from the cotton genomic region detected based on the synthesized amplicons during the virtual analysis process.

Using version 2.16.0 of the BLAST search tool [51], valuable candidate genes and proteins were identified among the probable genes and their transcript variants found with the assistance of the Augustus web application.

### 4.6. Statistical Analysis

To construct a high-resolution phylogenetic dendrogram, the multi-locus binary marker scoring matrix (0/1) was transformed into an aligned nucleotide format (0 to A; 1 to T) and analyzed via the Unweighted Pair Group Method with Arithmetic Mean (UPGMA) algorithm implemented in MEGA 11 software [52]. Quantitative agronomic traits and variance parameters were evaluated using one-way analysis of variance (One-Way ANOVA) via OriginPro software (version 2025; OriginLab Corp., Northampton, MA, USA). All agrobiometric data calculations strictly adhered to classical statistical methods for plant experimental design as outlined by Dospekhov (1985) [53].

## 5. Conclusions

The longstanding genetic stagnation observed in cotton breeding and the extreme narrowing of the gene pool of cultivated varieties necessitate the inclusion of wild allotetraploid resources in cotton improvement programs. In this study, when wild subspecies of *G. hirsutum* were hybridized with cultivated ssp. *euhirsutum* varieties, a high boll-setting coefficient (68.5–82.0%) and biological adaptability were observed; however, in remote interspecific hybridization, lower reproductive compatibility and marked photoperiod sensitivity were observed. These differences in reproductive compatibility were addressed by subjecting the plants to an artificial short-day regime of 10 h of light and 14 h of darkness, thereby promoting the transition of hybrid genotypes to the reproductive phase.

Cytogenetic observations showed a stable process of meiosis, characterized mainly by the predominance of bivalent pairing and a relatively low frequency of univalent and meiotic multivalents. These results indicate generally stable meiotic behavior, with no large-scale structural rearrangements directly demonstrated in the present study. In the F_2_ generation, epistatic and complementary genetic interactions may have contributed to the observed positive transgressive segregation for polygenic traits. However, these mechanisms were not directly tested in the present study and require further genetic investigation.

## Figures and Tables

**Table 1:** Hybridizations of *G. hirsutum* varietal diversities and the degree of fully developed seeds in hybrid bolls.

No.	Hybrid Combinations	Number of Hybridizations	Number of Hybrid Bolls Obtained	Percentage of Hybrid Bolls Set	Hybrid Bolls (F_0_)				
					Number of Analyzed Bolls	Seeds Set in Bolls, %			
						M ± SE	Range	SD	CV %
1	var. *m. palmerii ×* var. *nervosum (*V*)*	31	14	45.2	10	69.0 ± 2.04	62.7–75.7	1.41	6.44
2	var. *m. palmerii* × var. *nervosum* (Y)	36	17	47.2	10	64.7 ± 2.45	58.1–76.3	1.59	7.75
3	var. *m. palmerii ×* var. *java*	40	21	52.5	10	69.5 ± 2.68	60.4–74.5	1.87	8.48
4	var. *java* × var. *m. palmerii*	30	15	50.0	10	68.8 ± 2.31	62.4–76.6	1.59	7.30
5	var. *m. palmerii* × var. *el-salvador*	37	16	43.2	10	81.5 ± 2.44	65.7–88.2	1.99	7.72
6	var. *el-salvador* × var. *m. palmerii*	32	12	37.5	10	60.4 ± 4.19	51.2–74.3	2.53	13.23
7	var. *florida* × var. *hopi*	38	21	55.3	10	71.8 ± 2.47	64.7–85.4	1.78	7.82
8	var. *gambia* × var. *m. palmerii*	36	15	41.7	10	31.2 ± 4.79	26.3–38.7	1.50	15.14
9	var. *gambia* × ssp. *punctatum*	31	12	38.7	10	31.8 ± 2.54	28.4–34.8	0.81	8.03
10	var. *gambia* × ssp. *purpurascens*	30	11	36.7	10	41.7 ± 4.13	35.3–49.8	1.72	13.04
11	ssp. *paniculatum* × var. *hopi*	34	16	47.1	10	69.3 ± 2.72	62.8–78.9	1.89	8.61
12	ssp. *punctatum* × ssp. *purpurascens*	29	14	48.3	10	59.2 ± 2.89	52.1–66.1	1.71	9.14
13	ssp. *punctatum* × var. *m. galante*	35	18	51.4	10	72.7 ± 2.65	64.8–81.3	1.92	8.36
14	var. *m. galante* × ssp. *punctatum*	32	20	62.5	10	74.6 ± 1.89	70.3–82.1	1.41	5.97
15	var. *m. palmerii ×* AN-Bayavut 2	36	24	66.8	10	72.6 ± 2.03	63.7–78.2	1.48	6.42
16	AN-Bayavut 2 *×* ssp. *punctatum*	30	23	76.7	10	78.0 ± 2.11	72.4–88.4	1.65	6.68

Subsequent research aimed to determine the degree of interspecific hybridization among *G. hirsutum*, *G. barbadense*, and *G. darwinii*, as well as the rates of fully developed seeds in hybrid bolls.

**Table 2:** Genetic variation in boll weight in intraspecific and interspecific F_2_ hybrid populations.

No.	Hybrid Combinations	Samples	Classes K = 1					M ± SE	SD	CV, %
			<2	2.1–3.0	3.1–4.0	4.1–5.0	5<			
*G. hirsutum* × *G. hirsutum*										
1.	var. *m. palmerii ×* cv. AN-Bayavut 2	Number	-	54	78	66	-	3.6 ± 0.06	0.82	22.4
		%	-	27.3	39.4	33.3	-			
2.	cv. AN-Bayavut-2 *×* ssp. *punctatum*	Number	-	6	74	115	-	4.2 ± 0.04	0.58	13.8
		%	-	3.0	38.0	59.0	-			
3.	ssp. *punctatum* × var. *m. galante*	Number	-	4	44	147	2	4.4 ± 0.04	0.53	12.1
		%	-	2.0	22.3	74.6	1.1			
*G. hirsutum* × *G. barbadense*										
4.	var. *nervosum* (Y) *×* f. *brasiliense*	Number	-	78	77	45	-	3.4 ± 0.05	0.69	20.2
		%	-	39.0	38.5	22.5	-			
*G. hirsutum* × *G. darwinii*										
5.	*G. darwinii ×* var. *m. palmerii*	Number	59	110	31	-	-	2.4 ± 0.03	0.48	20.1
		%	29.5	55	15.5	-	-			
6.	*G. darwinii ×* var. *el-salvador*	Number	-	46	109	45	-	2.4 ± 0.03	0.47	19.6
		%	-	23.0	54.5	22.5	-			
*G. darwinii* × *G. barbadense*										
7.	*G. darwinii ×* f. *parnat*	Number	57	112	31	-	-	2.3 ± 0.03	0.46	20.2
		%	28.5	56.0	15.5	-	-			

**Table 3:** Taxonomic classification, designated abbreviations, and germplasm categories of the *Gossypium* taxa used in this study.

Species and Genome	Taxonomic Subspecies and Varieties	Designated Abbreviation	Germplasm Category	References
*Gossypium hirsutum* L. (AD_1_)	ssp. *mexicanum* var. *nervosum* (Yucatan)	var. *nervosum* (Y)	Wild form	Mauer F.M. (1954) [1]
	ssp. *mexicanum* var. *nervosum* (Victoria)	var. *nervosum* (V)	Wild form	Mauer F.M. (1954) [1]
	ssp. *mexicanum* var. *microcarpum palmerii*	var. *m. palmerii*	Wild form	Mauer F.M. (1954) [1]
	ssp. *punctatum*	ssp. *punctatum*	Exotic/Wild-type relative	Mauer F.M. (1954) [1]
	ssp. *punctatum* var. *java*	var. *java*	Exotic/Primitive	Mauer F.M. (1954) [1]
	ssp. *punctatum* var. *florida*	var. *florida*	Exotic/Primitive	Mauer F.M. (1954) [1]
	ssp. *punctatum* var. *hopi*	var. *hopi*	Exotic/Primitive	Mauer F.M. (1954) [1]
	ssp. *punctatum* var. *gambia*	var. *gambia*	Exotic/Primitive	Mauer F.M. (1954) [1]
	ssp. *paniculatum* (Rodesia)	ssp. *paniculatum (R)*	Exotic/Primitive	Mauer F.M. (1954) [1]
	ssp. *purpurascens* (red leaf)	ssp. *purpurascens*	Exotic/Primitive	Fryxell P.A. (1992) [6]
	ssp. *purpurascens* (green leaf)	ssp. *purpurascens* (2)	Exotic/Primitive	Fryxell P.A. (1992) [6]
	ssp. *purpurascens* var. *el-salvador*	var. *el-salvador*	Exotic/Primitive	Fryxell P.A. (1992) [6]
	ssp. *glabrum* var. *marie galante*	var. *m. galante*	Exotic/Primitive	Fryxell P.A. (1992) [6]
	ssp. *euhirsutum* cv. AN-Bayavut 2	cv. AN-Bayavut 2	Cultivated/Standard	
*Gossypium barbadense* L. (AD_2_)	ssp. *ruderale f. parnat*	f. *parnat*	Exotic/Primitive	Mauer F.M. (1954) [1]
	ssp. *ruderale f. ishan nigeria*	f. *i. nigeria*	Exotic/Primitive	Mauer F.M. (1954) [1]
	ssp. *vitifolium f. brasiliense (red stem)*	f. *brasiliense*	Exotic/Primitive	Mauer F.M. (1954) [1]
*Gossypium darwinii* Watt. (AD_5_)	Wild type species	*G. darwinii*	Wild	Fryxell P.A. (1992) [6]

## Data Availability

The data supporting the findings of this study are available within the article and its Supplementary Materials.

## Supplementary Materials

The following supporting information can be downloaded at: https://www.mdpi.com/article/10.3390/plants15182866/s1, Table S1. Indicators of hybridization and fully developed seed production in interspecific crosses. Table S2. Inheritance and variability of boll weight (g) and fiber length (mm) in intraspecific and interspecific F_1_ hybrid progenies. Table S3. Inheritance of fiber yield, 1000-seed weight in intraspecific and interspecific F_1_ hybrids. Table S4. Variability of fiber length in interspecific and intraspecific F_2_ hybrid progeny. Table S5. Variability of the fiber yield in intra- and interspecific F_2_ progenies. Table S6. Variability range of the 1000-seed weight in intra- and interspecific F_2_ hybrid combinations. Table S7. Cytogenetic analysis of chromosome conjugation at metaphase I of F_1_ hybrids. Table S8. Spore analysis and meiotic index in cotton F_1_ hybrids. Table S9. Pollen fertility measurements in cotton F_1_ hybrids. Table S10. Genetic differentiation parameters of the genotypes by the DNA markers. Table S11. Results of in-silico PCR analysis for the flanking regions of polymorphic SSR markers in the genome of tetraploid cotton species. Table S12. Information on putative genes and their transcripts identified in the marked regions using the AUGUSTUS web application. Table S13. Putative genes identified in marker regions and the proteins they encode by means of BLAST analysis. Figure S1. Variation and distribution of fiber length (mm) across parental genotypes (P_1_, P_2_) and hybrid populations (F_1_, F_2_) in seven cotton cross combinations. Boxplots show the interquartile range (IQR, 25–75th percentiles) with the median indicated by the horizontal line inside the box. Red diamonds with numerical labels represent population means (M). Green labels indicate maximum values, purple labels indicate minimum values, and individual dots represent single plant observations. Figure S2. Variation and distribution of fiber yield (%) across parental genotypes (P_1_, P_2_) and hybrid populations (F_1_, F_2_) in seven cotton cross combinations. Boxplots show the interquartile range (IQR, 25–75th percentiles) with the median indicated by the horizontal line inside the box. Red diamonds with numerical labels represent population means (M). Green labels indicate maximum values, purple labels indicate minimum values, and individual dots represent single plant observations. Figure S3. Variation and distribution of thousand seed-weight (g) across parental genotypes (P_1_, P_2_) and hybrid populations (F_1_, F_2_) in seven cotton cross combinations. Boxplots show the interquartile range (IQR, 25–75th percentiles) with the median indicated by the horizontal line inside the box. Red diamonds with numerical labels represent population means (M). Green labels indicate maximum values, purple labels indicate minimum values, and individual dots represent single plant observations. Figure S4. Similarity dendrogram constructed based on gene-targeted and SSR markers of the studied genotypes.
