# Supplementary material for: Genetic Dynamics of Allotetraploid Cotton Species (Gossypium spp.) and Their Utilization in Breeding for Superior Fiber Quality
Source: Plants (Basel). 2026 Sep 19;15(18):2866. doi: 10.3390/plants15182866 (PMC13611794; doi:10.3390/plants15182866)
Supplement: Supplementary file 1 [file plants-15-02866-s001.zip › plants-4520098-supplementary.pdf]

Table S1. Indicators of hybridization and fully developed seed production in interspecific crosses.

No.	Hybrid combinations	Number of hybridizations	Number of hybrid bolls	Percentage of hybrid bolls set, %	Number of analyzed bolls	Hybrid bolls (F_0)			
						Seeds set in bolls, %			
						M ± SE	Range	SD	CV %
				G. hirsutum × *G. barbadense*					
1	f. *i. nigeria* × var. *hopi*	25	14	56.0	10	49.2±6.65	36.2-66.6	3.27	21.01
2	var. *nervosum* (Y) × f. *i. nigeria*	22	10	45.5	10	51.1 ± 4.02	43.5-62.2	2.05	12.69
3	f. *i. nigeria* × var. *nervosum* (Y)	20	9	45.0	9	45.9 ± 4.05	38.7-55.6	1.86	12.80
4	var. *nervosum* (Y) × f. *brasiliense*	18	10	55.6	10	46.6±5.43	40.3-60.4	2.53	5.43
5	f. *brasiliense* × var. *nervosum* (Y)	21	11	52.4	10	38.8±4.11	32.4-45.7	1.60	12.99
				G. hirsutum × *G. darwinii*					
6	*G. darwinii* × var. *m.palmerii*	21	8	38.1	8	45.0 ± 9.67	41.3 - 56.7	1.62	11.34
7	*G. darwinii* × var. *hopi*	17	5	29.4	5	43.0±3.50	39.2-53.5	1.51	11.06
8	*G. darwinii* × var. *el-salvador*	16	4	25.0	4	44.2±3.55	38.4-52.1	1.57	11.23
9	var. *el-salvador* × *G. darwinii*	22	7	31.8	7	42.2±4.31	34.8-53.5	1.82	13.63
				G. barbadense × *G. darwinii*					
10	*G. darwinii* × f. *parnat*	18	6	33.3	6	90.4±0.88	87.6-94.5	0.80	2.78
11	f. *parnat* × *G. darwinii*	20	12	60	10	83.5±1.22	80.2-88.4	0.91	3.87
12	*G. darwinii* × f. *brasiliense*	17	12	70	10	82.3±0.94	80.2-87.6	0.78	2.98
13	f. *brasiliense* × *G. darwinii*	19	12	63.2	10	73.5±1.53	71.2-80.4	1.12	4.83

Table S2. Inheritance and variability of boll weight (g) and fiber length (mm) in intraspecific and interspecific F_1 hybrid progenies

№	Parents and hybrid generations	Boll weight(g)			hp	Fiber length, mm			hp
		M±SE	Range	CV%		M±SE	Range	CV%	
	G. hirsutum								
1	var. *nervosum* (V)	1.00±0.04	0.80-1.10	13.33	-	22.6±1.98	21-25	6.26	
2	var. *nervosum* (Y)	1.10±0.04	1.00-1.30	10.93	-	25.8±1.13	24-27	3.56	
3	var. *m. palmerii*	1.06±0.04	0.80-1.20	11.93	-	23.0±1.72	21-25	5.42	
4	var. *java*	1.02±0.04	0.80-1.20	12.05	-	27.2±1.53	26-30	4.84	
5	var. *florida*	1.36±0.05	1.40-1.80	11.60	-	26.1±2.25	24-30	7.10	
6	var. *gambia*	1.50±0.04	1.40-1.80	8.82	-	28.3±2.36	24-30	7.07	
7	var. *hopi*	1.32±0.06	1.20-1.50	11.32	-	22.2±1.98	20-24	6.26	
8	ssp. *punctatum*	3.30±0.14	3.30-4.20	13.25	-	25.0±1.91	22-27	6.05	
9	ssp. *paniculatum*	2.50±0.14	1.80-2.80	17.20	-	28.5±2.15	26-30	6.79	
10	cv. AN-Bayaut 2	5.10±0.10	4.50-5.50	6.37	-	32.7±1.21	31-34	3.83	
11	ssp. *purpurascense*	3.80±0.09	3.60-4.00	7.55	-	28.1±1.41	27-30	4.44	
12	ssp. *purpurascens* (2)	3.60±0.09	3.40-4.10	14.65	-	30.4±1.66	27-31	5.25	
13	var. *el-salvador*	1.90±0.09	1.70-2.40	14.57	-	25.2±2.50	22-29	7.89	
14	var. *m.galante*	3.50±0.10	3.10-4.10	9.20	-	34.8±0.72	34-36	2.27	
	G. barbadense								
15	f. *parnat*	1.60±0.09	1.40-2.30	17.92	-	21.6 ±2.31	19-24	7.30	
16	f. *i. nigeria*	1.60±0.09	1.40-2.20	15.50	-	23.7 ±2.00	21-26	6.31	
17	f. *brasiliense*	2.70±0.18	1.80-2.50	20.51	-	25.5 ± 2.05	22-27	6.47	
	G. darwinii								
18	*G. darwinii*	2.3±0.21	1.90-2.80	20.78	-	38.4±1.70	34-41	5.38	
	G. hirsutum × *G. hirsutum* (F_1)								
19	var. *m. palmerii* × var. *nervosum* (V)	1.40±0.04	1.20-1.60	8.78	12.33	25.4 ± 2.05	23-28	6.48	13
20	var. *m. palmerii* × var. *nervosum* (Y)	1.38±0.03	1.20-1.50	6.83	5.0	25.3 ± 1.34	24-28	4.19	0.64
21	var. *m. palmerii* × var. *java*	1.30±0.04	1.20-1.50	0.51	13.0	28.2 ± 1.8	26-30	5.74	1.47
22	var. *java* × var. *m.palmerii*	1.40±0.04	1.20-1.60	9.82	18.0	29.0±0.31	27-30	3.61	1.86
23	var. *florida* × var. *hopi*	1.70±0.04	1.40-1.80	7.59	7	25.0±0.44	22-28	5.56	0.44
24	var. *gambia* × var. *m. palmerii*	1.80±0.04	1.60-2.00	7.64	2.5	30.1±0.86	26-31	2.72	3.45
25	var. *gambia* × ssp. *punctatum*	2.10±0.06	1.90-2.40	8.98	-0.3	30.3 ± 0.86	29-31	2.72	2.21
26	ssp. *paniculatum* × var. *hopi*	1.50±0.02	1.40-1.60	4.44	-0.8	25.9 ± 1.34	25-28	4.25	0.17
26	var. *m.palmerii* × AN-Bayaut 2	3.00±0.13	2.50-3.70	13.47	-0.05	32.2 ±1.95	29-34	6.18	0.89
27	AN-Bayavut 2 × ssp. *punctatum*	4.90±0.15	4.40-5.80	9.88	0.7	33.7 ± 0.99	34-35	3.14	1.26
28	var. *m. palmerii* × var. *el-salvador*	2.00±0.09	1.60-2.50	14.24	1.2	32.0±0.66	29-34	6.58	6.94
29	var. *el-salvador* × var. *m.palmerii*	2.20±0.09	1.80-2.70	13.38	1.7	28.1±2.22	26-31	7.01	3.64
30	ssp. *punctatum* × ssp. *purpurascens*	4.00±0.07	23.7-25.4	5.53	3.6	31.4±0.47	29-34	4.81	3.90
31	ssp. *punctatum* × var. *m.galante*	4.00±0.08	3.70-4.40	6.67	6.0	32.8±1.10	31-34	3.46	0.59
32	var. *m.galante* × ssp. *punctatum*	4.20±0.09	3.80-4.50	6.45	8.0	33.3±0.90	32-34	2.85	0.69
	G. hirsutum × *G. barbadense*								
33	f. *i. nigeria* × var. *hopi*	1.70±0.02	1.70-1.80	4.38	1.7	26.0±1.41	25-28	4.44	4.06
34	var. *nervosum* (Y) × f. *i. nigeria*	1.60±0.05	1.40-1.80	9.55	1.0	26.5±1.41	25-28	4.45	1.66
35	f. *i. nigeria* × var. *nervosum* (Y)	1.70±0.02	1.60-1.80	4.38	1.4	25.8±1.51	25-28	4.76	1
36	var. *nervosum* (Y) × f. *brasiliense*	3.00±0.16	2.40-3.90	17.32	1.4	26.4 ±2.05	25-29	6.49	5
37	f. *brasiliense* × var. *nervosum* (Y)	3.30±0.07	2.90-3.50	6.62	1.7	27.0 ±1.56	25-29	4.49	2.21
	G. hirsutum × *G. darwinii.*								
38	*G. darwinii* Watt. × var. *m. palmerii*	1.80±0.10	1.60-2.50	17.95	0.2	38.7 ± 1.95	36-41	6.18	1.04

39	*G. darwinii* Watt. × var. *hopi*	2.30±0.11	2.00-2.70	14.92	1.0	39.4 ±1.43	37-41	4.51	1.12
40	*G. darwinii* Watt. × var. *el-salvador*	2.50±0.06	2.20-2.70	7.06	2.0	38.8 ± 1.14	37-41	3.60	1.06
41	var. *el-salvador* × *G. darwinii*	2.40±0.04	2.30-2.60	5.20	1.5	38.3 ±0.68	37-39	2.15	0.98
	G. darwinii × *G. barbadense*								
42	*G. darwinii* × f.*parnat*	2.40±0.04	2.30-2.60	5.20	1.3	36.4 ± 2.40	33-40	7.57	0.76
43	f. *parnat* × *G. darwinii*	1.90±0.04	1.70-2.10	7.22	-0.14	36.1 ± 1.20	34-38	3.80	0.73
44	*G. darwinii* × f. *brasiliense*	3.10±0.04	2.90-3.40	4.30	3.0	39.7 ± 1.07	37-41	3.37	1.20
45	f. *brasiliense* × *G. darwinii*	3.00±0.03	2.80-3.10	3.69	2.5	37.5 ± 0.82	36-39	2.59	0.86

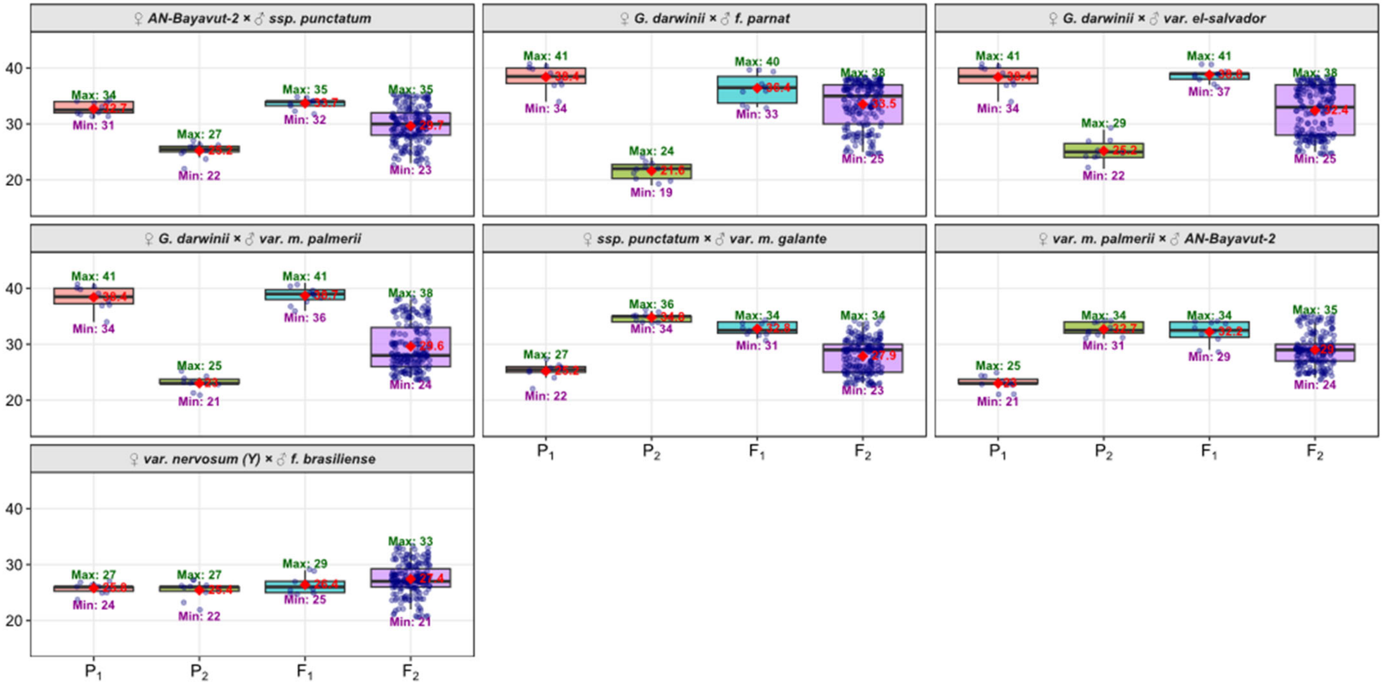

Figure S1. Variation and distribution of fiber length (mm) across parental genotypes (P_1, P_2) and hybrid populations (F_1, F_2) in seven cotton cross combinations. Boxplots show the interquartile range (IQR, 25–75th percentiles) with the median indicated by the horizontal line inside the box. Red diamonds with numerical labels represent population means (M). Green labels indicate maximum values, purple labels indicate minimum values, and individual dots represent single plant observations.

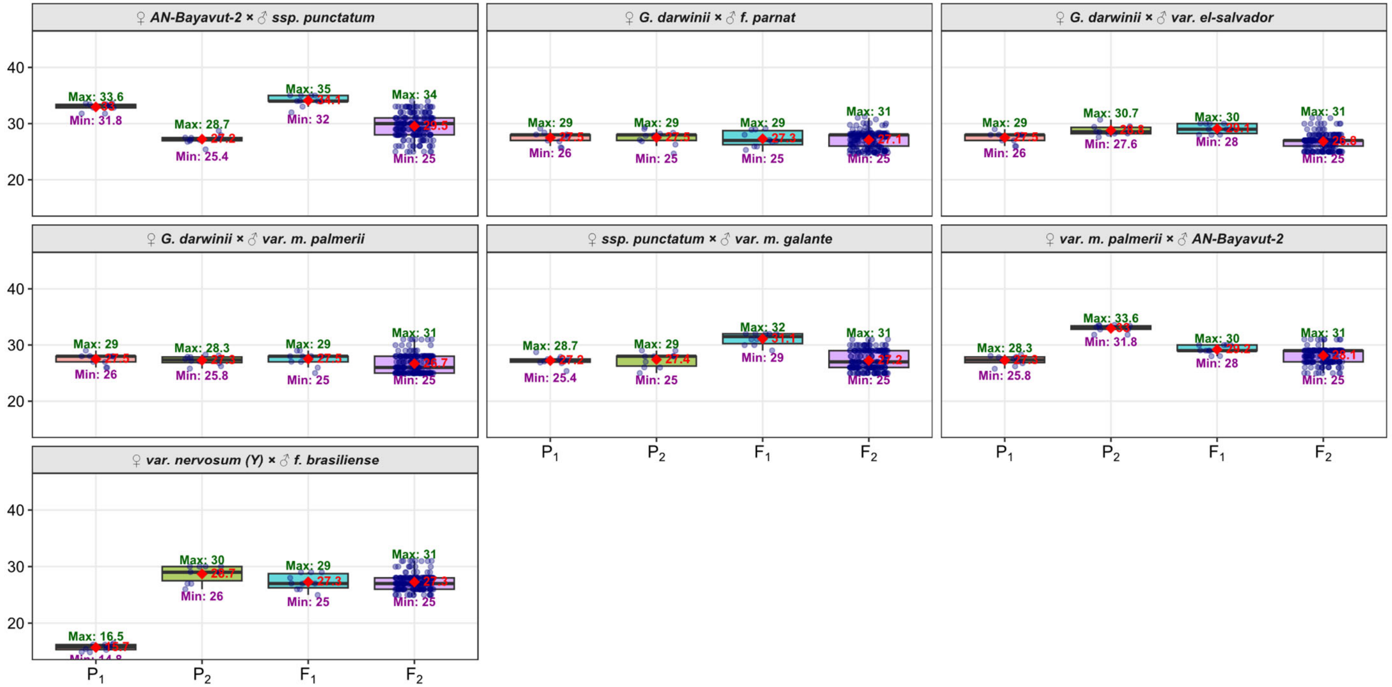

Figure S2. Variation and distribution of fiber yield (%) across parental genotypes (P_1, P_2) and hybrid populations (F_1, F_2) in seven cotton cross combinations. Boxplots show the interquartile range (IQR, 25–75th percentiles) with the median indicated by the horizontal line inside the box. Red diamonds with numerical labels represent population means (M). Green labels indicate maximum values, purple labels indicate minimum values, and individual dots represent single plant observations.

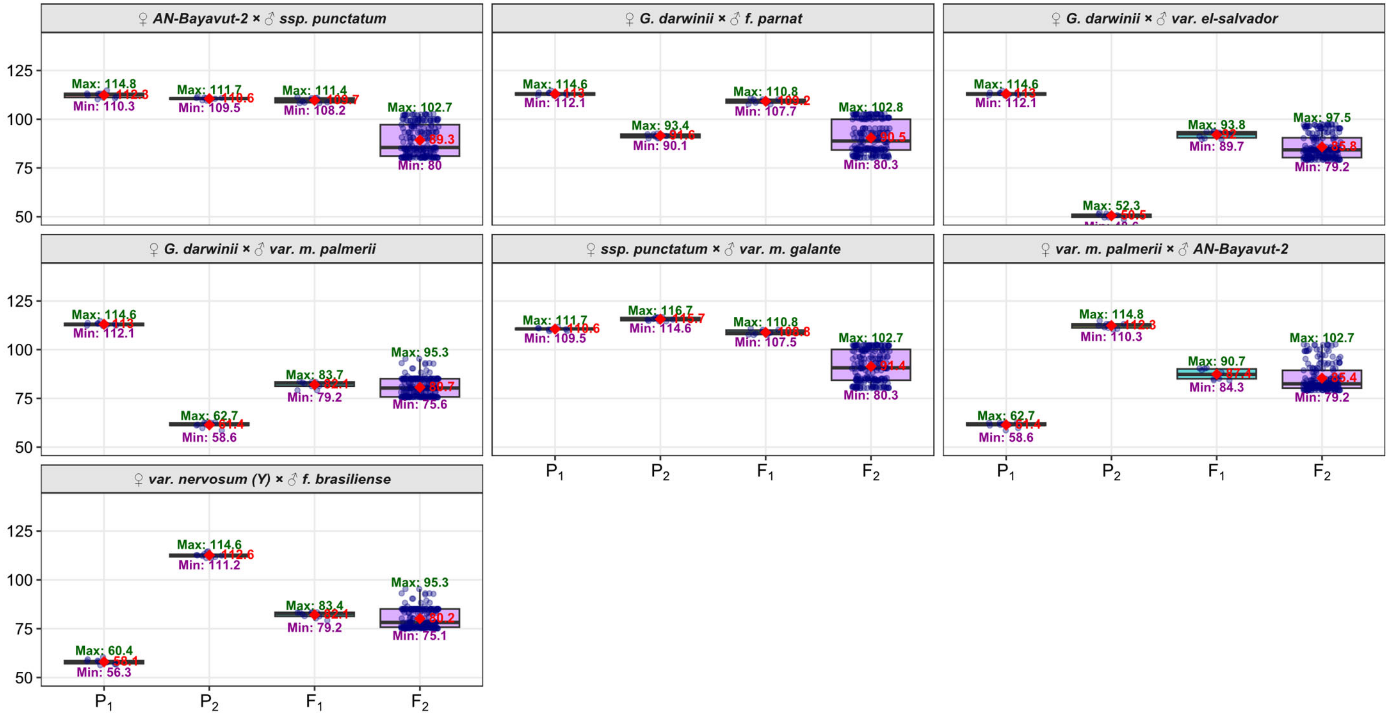

Figure S3. Variation and distribution of thousand seed-weight (g) across parental genotypes (P_1, P_2) and hybrid populations (F_1, F_2) in seven cotton cross combinations. Boxplots show the interquartile range (IQR, 25–75th percentiles) with the median indicated by the horizontal line inside the box. Red diamonds with numerical labels represent population means (M). Green labels indicate maximum values, purple labels indicate minimum values, and individual dots represent single plant observations.

Table S3. Inheritance of fiber yield, 1000-seed weight in intraspecific and interspecific F_1 hybrids.

№	Parent genotypes and hybrid generations	Fiber yield, %				Thousand Seed Weight (g)			
		M±SE	Range	CV%	hp	M±SE	Range	CV%	hp
				G. hirsutum					
1	var. *nervosum* (V)	26.7±1.00	25-28	3.17	-	60±0.63	58.4-61.8	1.98	
2	var. *nervosum* (Y)	15.7±1.19	14-16	3.75	-	58.1±0.68	56.3-60.4	2.14	
3	var. *m. palmerii*	27.3±0.95	25-28	3.00	-	61.4±0.68	58.6-62.7	2.14	
4	var. *java*	22.2±1.56	20-23	4.93	-	65.8±0.71	63.8-68.2	2.25	
5	var. *florida*	23.0±2.15	20-26	6.80	-	53.2±0.78	51.6-55.2	2.46	
6	var. *gambia*	33.3±0.68	30-34	2.14	-	110.5±0.44	107-111.8	1.40	
7	var. *hopi*	18.2±0.96	17-19	3.04	-	83.4±0.76	80.5-87.0	2.41	
8	ssp. *punctatum*	27.2±0.98	25-29	3.11	-	110.6±0.19	109.5-111.7	0.60	
9	ssp. *paniculatum*	28.5±0.92	27-30	2.89	-	78.3±0.57	75.6-80.2	1.80	
10	cv. AN-Bayavut 2	33.0±0.65	31-34	2.04	-	112.3±0.23	114.6-116.7	0.73	
11	ssp. *purpurascens*	31.0±1.01	30-32	3.18	-	85.2±0.31	84.2-86.3	0.97	
12	ssp. *purpurascens* (2)	30.0±0.64	29-31	2.01	-	85.5±0.36	84.2-87.5	1.15	
13	var. *el-salvador*	28.5±0.67	28-29	2.13	-	50.5±0.68	48.6-52.3	2.14	
14	var. *m. galante*	27.4±1.56	25-29	4.93		115.7±0.23	114.6-116.7	0.73	
				G. barbadense					
15	f. *parnat*	27.5 ±1.46	27-29	4.62	-	91.5±0.39	90.1-93.4	1.24	
16	ssp. *ruderale* f. *ishan nigeria*	25.3 ±2.51	24-27	7.92	-	119±0.39	115.7-121.5	1.75	
17	ssp. *vitifolium* f. *brasiliense*	28.7±1.65	26-30	5.21	-	112.6±0.32	112.2-114.6	1.01	
				G. darwinii					
18	*G. darwinii*	27.5±1.12	26-29	3.53	-	113±0.22	112.1-114.6	0.69	
				G. hirsutum × *G. hirsutum*					
19	var. *m. palmerii* × var. *nervosum* (V)	25.3±1.19	24-27	3.75	-2.8	93.5±0.42	92.1-95.3	1.32	46.86
20	var. *m. palmerii* × var. *nervosum* (Y)	25.2±1.30	24-27	4.10	0.65	93.7±0.39	92.1-95.3	1.23	20.57
21	var. *m. palmerii* × var. *java*	24.6±0.90	24-26	2.84	-0.04	85±0.35	83.5-86.4	1.10	9.72
22	var. *java* × var. *m. palmerii*	25.4±0.33	24-27	4.08	0.28	85.1±0.33	84.2-86.4	1.05	9.77
23	var. *florida* × var. *hopi*	26.4±0.25	25-28	2.98	2.39	98.5±0.31	96.4-99.8	0.97	2
24	var. *gambia* × var. *m. palmerii*	29.4±0.43	26-31	4.60	-0.28	122.3±0.45	119.5-124.4	1.41	1.48
25	var. *gambia* × ssp. *punctatum*	30.7±0.98	29-32	3.09	0.15	111.2±0.16	110.3-112.1	0.50	13
26	ssp. *paniculatum* × var. *hopi*	26.1±1.56	25-27	4.93	0.52	84.5±1.56	82.1-87.3	4.93	1.44
27	var. *m. palmerii* × AN-Bayavut-2	29.2±0.85	28-30	2.70	-0.31	87.4±1.51	84.3-90.7	4.76	0.02
28	AN-Bayavut 2 × ssp. *punctatum*	34.1±0.92	32-35	2.92	1.38	109.7±0.38	108.2-111.4	1.19	-2.05
29	var. *m. palmerii* × var. *el-salvador*	35.0±0.30	34-36	2.71	11.00	83±0.52	80.5-85.4	1.64	4.96
30	var. *el-salvador* × var. *m. palmerii*	29.2±1.97	26-31	6.21	2.08	82.1±0.54	80.1-84.2	1.69	4.79
31	ssp. *punctatum* × ssp. *purpurascens*	24.6±0.21	24-25	2.66	-2.68	120±0.29	118.6-121.8	0.92	1.74
32	ssp. *punctatum* × var. *m.galante*	31.1±1.12	29-32	3.54	38	108.8±0.35	107.5-110.8	1.12	-1.70
33	var. *m.galante* × ssp. *punctatum*	31.6±1.51	30-32	4.76	0.69	110±0.28	108.4-110.9	0.89	-1.23
				G. hirsutum × *G. barbadense*					
34	f. *i. nigeria* × var. *hopi*	24.9±0.94	24-26	2.96	0.89	90±0.59	86.4-91.4	1.86	3.88
35	var. *nervosum* (Y) × f. *i. nigeria*	25.3±1.19	24-25	3.75	1	81±0.47	79.2-82.5	1.47	0.63
36	f. *i. nigeria* × var. *nervosum* (Y)	24.0±1.39	23-26	4.39	0.73	79.7±0.46	78.3-81.3	1.45	0.54
37	var. *nervosum* (Y) × f. *brasiliense*	27.3±1.64	25-29	5.19	0.78	82.1±0.58	79.2-83.4	1.83	-0.11
38	f. *brasiliense* × var. *nervosum* (Y)	27.8 ±1.05	26-29	3.31	0.86	91±0.41	89.7-93.7	1.31	0.20
				G. hirsutum × *G. darwinii.*					
39	*G. darwinii* × var. *m. palmerii*	27.5 ± 1.46	25-29	4.62	1	82 ±0.67	79.2-83.7	2.11	-0.20
40	*G. darwinii* × var. *hopi*	28.0 ±0.92	27-29	2.92	1.11	84±0.50	82.3-85.7	1.58	-0.95
41	*G. darwinii* × var. *el-salvador*	29.1 ± 0.95	28-30	3.01	2.20	92±0.58	89.7-93.8	1.83	0.32
42	var. *el-salvador* × *G. darwinii*	28.8 ±1.01	28-30	3.19	1.6	83.2±0.40	81.3-85.1	1.26	0.04
				G. darwinii × *G. barbadense*					

43	*G. darwinii* × f. *parnat*	27.3 ± 1.54	25-29	7.57	-1.66	109.2 ±0.33	107.7-110.8	1.03	0.64
44	f. *parnat* × *G. darwinii*	28.4 ± 1.20	26-29	3.79	2	93.2±0.39	91.2-94.7	1.24	-0.84
45	*G. darwinii* × f. *brasiliense*	30.5 ± 1.71	29-32	5.41	4	114±0.32	112.2-115.4	1.00	6
46	f. *brasiliense* × *G. darwinii*	28.7 ± 1.56	36-39	4.94	1	113.2±0.34	111.7-115.4	1.09	2

Table S4. Variability of fiber length in interspecific and intraspecific F_2 hybrid progeny.

No.	Hybrid combinations	Number of plants	Classes, K=3						M±SE	SD	CV, %
			23.0-25.9	26.0-28.9	29.0-31.9	32.0-34.9	35.0-37.9	38.0-40.9			h^2
				G. hirsutum × *G. hirsutum*							
1.	var. *m. palmerii* × cv. AN-Bayaut-2	number	-	39	96	32	30	-	29.0±0.21	2.92	10.8
		%	-	19.8	48.7	16.2	15.2	-			
2.	cv. AN-Bayavut 2 × ssp. *punctatum*	number	5	31	58	59	42	-	29.6±0.23	3.21	10.86
		%	2.5	15.9	29.7	30.2	21.5	-			
3.	ssp. *punctatum* × var. *m. galante*	number	-	17	60	65	54	8	27.8±0.20	2.92	10.51
		%	-	8.3	29.4	31.9	26.5	3.92			
				G. hirsutum × *G. barbadense*							
4.	var. *nervosum* (Y) × f. *brasiliense*	number	16	45	89	43	7	-	27.0±0.19	2.66	9.84
		%	8.0	22.5	44.5	21.5	3.5	-			
				G. hirsutum × *G. darwinii*							
5.	*G. darwinii* × var. *m. palmerii*	number	30	74	28	20	37	11	30.0±0.29	4.15	13.84
		%	15.0	37.0	14.0	10.0	18.5	5.5			
6.	*G. darwinii* × var. *el-salvador*	number	9	57	17	32	77	8	32.4±0.30	4.25	13.12
		%	4.5	28.5	8.5	16.0	38.5	4.0			
				G. darwinii × *G. barbadense*							
7.	*G. darwinii* × f. *parnat*	number	9	29	17	36	100	9	33.5±0.29	4.03	12.04
		%	4.5	14.5	8.5	18	50.0	4.5			

Table S5. Variability of the fiber yield in intra- and interspecific F_2 progenies

No.	Hybrid combinations	Number of plants	Classes, K=2					M±SE	SD	CV, %
			25.0-26.9	27.0-28.9	29.0-30.9	31.0-32.9	33.0-34.9			
			G. hirsutum × *G. hirsutum*							
1.	ssp. var. *m. palmerii* × cv. AN-Bayavut 2	number	21	76	91	9	-	28.1±0.29	1.15	4.8
		%	10.6	38.2	46.1	4.6	-			
2.	cv. AN-Bayavut 2 × ssp. *punctatum*	number	19	42	72	41	21	29.5±0.52	2.23	7.23
		%	9.7	21.5	36.9	21.0	10.8			
3.	ssp. *punctatum* × var. *m. galante*	number	88	52	60	4	-	27.2±0.44	1.69	6.23
		%	43.1	25.5	29.4	2	-			
			G. hirsutum × *G. barbadense*							
4.	var. *nervosum* (Y) × f. *brasiliense*	number	57	122	13	8	-	27.2±0.35	1.34	4.92
		%	28.5	61.0	6.5	4	-			
			G. darwinii × *G. hirsutum*							
5.	*G. darwinii* × *G* var. *m. palmerii*	number	102	80	11	7	-	26.6±0.43	1.61	6.07
		%	51.0	40.0	5.5	3.5	-			
6.	*G. darwinii* × var. *el-salvador*	number	66	117	14	3	-	26.6±0.36	1.37	5.10
		%	33.0	58.5	7.0	1.5	-			
			G. darwinii × *G. barbadense*							
7.	*G. darwinii* × f. *parnat*	number	70	113	14	3	-	27.1±0.39	1.45	5.45
		%	35.0	56.0	7.0	1.5	-			

Table S6. Variability range of the 1000-seed weight in intra- and interspecific F_2 hybrid combinations.

No.	Hybrid combinations	Number of plants	Classes, K=5						M±SE	SD	CV, %
			75.0-79.9	80.0-84.9	85.0-89.9	90.9-94.9	95.0-99.9	100.0-104.9			
			G. hirsutum × G. hirsutum								
1.	var. *m. palmerii* × cv. AN-Bayavut-2	number	30	98	19	20	15	15	85.5±0.66	7.17	8.78
		%	15.2	49.7	9.6	10.1	7.6	7.6			
2.	AN-Bayavut 2 × ssp. *punctatum*	number	-	81	26	25	34	29	89.3±0.64	8.00	8.9
		%	-	41.5	13.3	12.8	17.4	14.9			
3.	ssp. *punctatum* × var. *m. galante*	number	-	63	26	25	33	50	91.4±0.64	8.17	8.94
		%	-	40.0	13.2	12.7	16.7	25.4			
			G. hirsutum × G. barbadense								
4.	var. *nervosum* (Y) × f. *brasiliense*	number	112	26	53	7	2	-	80.1±0.45	5.09	6.36
		%	56.0	13.0	26.5	3.5	1.0	-			
			G. hirsutum × G. darwinii								
5.	*G. darwinii* × var. *m. palmerii*	number	94	43	54	7	2	-	80.7±0.42	4.84	5.99
		%	47.0	21.5	27.0	3.5	1.0	-			
6.	*G. darwinii* × var. *el-salvador*	number	22	99	23	20	36	-	85.8±0.50	6.11	7.12
		%	11.0	49.0	11.5	10.0	18.0	-			
			G. darwinii × G. barbadense								
7.	*G. darwinii* × f. *parnat*	number	-	74	29	30	11	56	90.5±0.63	8.01	8.85
		%	-	37.0	14.5	15.0	5.5	28.0			

Table S7. Cytogenetic analysis of chromosome conjugation at metaphase I of F_1 hybrids.

No.	Hybrid combination	Cells analyzed (n)	Univalents (I)	Bivalents (II)	Quadrivalents (IV)
			G. hirsutum × G. hirsutum		
1	var. *m. palmerii* × AN-Bayavut-2	50	0.4 ± 0.1	25.8 ± 0.2	0.0
2	AN-Bayavut 2 × ssp. *punctatum*	50	0.7 ± 0.2	25.5 ± 0.3	0.1 ± 0.1
			G. hirsutum × G. barbadense		
3	f. *i. nigeria* × var. *hopi*	52	0.42±0,16	25.79±0.08	0.0
4	var. *nervosum* (Y) × f. *brasiliense*	40	0.15±0.08	25.92±0.04	0.0
			G. hirsutum × G. darwinii		
5	*G. darwinii* × var. *el-salvador*	54	0.18±0.05	25.63±0.11	0.0
6	var. *el-salvador* × *G. darwinii*	43	0.21±0.06	25.58±0.12	0.0
			G. barbadense × G. darwinii		
7	*G. darwinii* × f. *brasiliense*	47	0.0	25.70±0.12	0.13±0.06
8	f. *brasiliense* × *G. darwinii*	40	0.0	25.80±0.10	0.10±0.05

Table S8. Spore analysis and meiotic index in cotton F_1 hybrids

№	Hybrid combination	Total number of spores (n)	Anomalous sporads (<4 microspores), %	Meiotic index, %	Tetrads with micronuclei, %	Anomalous sporads (>4 microspores), %
				G. hirsutum × G. hirsutum		
1.	var. *m.palmerii* × AN-Bayavut 2	1118	9.84±0.89	90.16±0.89	0.0	0.0
2.	AN-Bayavut 2 × ssp. *punctatum*	1320	8.79±0.78	91.21±0.78	0.0	0.0
				G. hirsutum × G. barbadense		
3.	f. *i. nigeria* × var. *hopi*	1484	10.71±0.80	84.10±0.95	1.89±0.35	3.30±0.46
4.	var. *nervosum* (Y) × f. *brasiliense*	1399	9.63±0.70	84.20±0.98	1.93±0.37	3.29±0.48
				G. hirsutum × G. darwinii		
5.	*G. darwinii* × var. *el-salvador*	1131	8.13±0.81	87.00±1.00	1.95±0.41	2.92±0.50
6.	var. *el-salvador* × *G. darwinii*	1161	8.27±0.81	86.48±1.00	2.15±0.43	3.10±0.51
				G. barbadense × G. darwinii		
7.	*G. darwinii* × f. *brasiliense*	823	10.21±1.06	85.66±1.22	0.0	4.13±0.69

8.	f. *brasiliense* × *G. darwinii*	861	11.50±1.09	84.32±1.24	0.0	4.18±0.68

Table S9. Pollen fertility measurements in cotton F_1 hybrids

№	Hybrid combination	Number of flowers analyzed (n)	Total number of pollen grains (n)	Pollen fertility, %
	G. hirsutum × *G. hirsutum*			
1.	var. *m.palmerii* × AN-Bayavut-2	4	2590	90.82±0.57
2.	AN-Bayavut-2 × ssp. *punctatum*	4	1904	94.12±0.54
	G. hirsutum × *G. barbadense*			
3.	f. *i. nigeria* × var. *hopi*	4	2714	82.42±0.73
4.	var. *nervosum* (Y) × f. *brasiliense*	4	2365	83.04±0.77
	G. hirsutum × *G. darwinii*			
5.	*G. darwinii* × var. *el-salvador*	4	1859	81.66±0.90
6.	var. *el-salvador* × *G. darwinii*	4	1876	82.14±0.88
	G. barbadense × *G. darwinii*			
7.	*G. darwinii* × f. *brasiliense*	4	2709	84.05±0.70
8.	f. *brasiliense* × *G. darwinii*	4	2578	84.52±0.71

Table S10. Genetic differentiation parameters of the genotypes by the DNA markers

No.	Marker name	Number of alleles	Heterozygosity (He)	Polymorphism information content (PIC)
1	POD2	2	0.50	0.375
2	NAU3284	2	0.50	0.375
3	CAT	7	0.59	0.5663
4	NAU5212	4	0.75	0.703
5	SOC 1 1	11	0.90	0.8882
6	RBOH	2	0.50	0.3743
7	PP2C78	3	0.70	0.5923
8	FLD2	2	0.50	0.3749
9	GR_318	2	0.50	0.375
10	CYP707A	3	0.70	0.5925
11	NCED1	2	0.50	0.3732
12	PSY	3	0.70	0.5925

No.	Marker name	Number of alleles	Heterozygosity (He)	Polymorphism information content (PIC)
13	PIF4_ 2	2	0.50	0.3748
14	BCH	4	0.56	0.5219
15	BNL1122	2	0.50	0.3749
16	PP2C8	9	0.89	0.8777
17	PYL4	2	0.50	0.3749
18	NHX2	7	0.86	0.8372
19	GST	2	0.50	0.375
20	BNL0840	7	0.86	0.8382
21	BNL3601	6	0.83	0.8095
22	BNL3255	14	0.93	0.9225
23	BNL3545	7	0.86	0.837
24	BNL4108	5	0.80	0.7677
25	BNL2655	3	0.70	0.5925

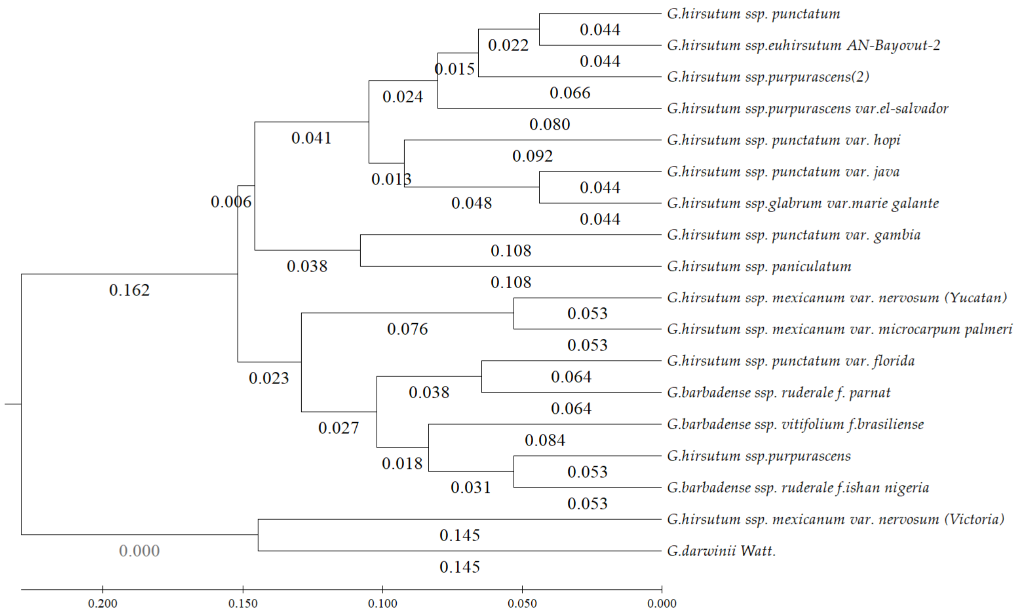

Figure S4. Similarity dendrogram constructed based on gene-targeted and SSR markers of the studied genotypes.

Table S11. Results of *in-silico* PCR analysis for the flanking regions of polymorphic SSR markers in the genome of tetraploid cotton species.

No.	Marker Name	Chromosomal Location	Genomic Coordinates of the Virtual Amplicon	Amplicon Size (bp)	Gene-Flanking Region Boundaries (−50 kb upstream / +50 kb downstream)
			G. hirsutum		
1.	NAU 3478	Dt_chr11	22 515 533 - 22 615 720	188	22 465 533 - 22 665 720
2.	NAU 3284	chr. 26	5 458 922 - 5 559 078	157	5 408 922 - 5 609 078
		chr. 21	13 411 450 - 13 511 604	155	13 361 450 - 13 561 604
3.	NAU 2951	chr. 21	4 244 673 - 4 344 838	166	4 194 673 - 4 394 838
		chr. 26	16 237 998 - 16 338 163	166	16 187 998 - 16 388 163
4.	NAU 5212	Dt_chr11	22 873 683 - 22 973 947	265	22 823 683 - 23 023 947
5.	BNL 3255	scaffold149.1	1 543 371 - 1 643 601	231	1 493 371 - 1 693 601
6.	BNL 1604	chr. 9	80 264 814 - 80 364 934	121	80 214 814 - 80 414 934
7.	BNL 1122	chr. 9	90 454 059 - 90 554 232	174	90 454 059 - 90 604 232
			G. barbadense		
1.	NAU 3478	chr. D_{11}	21 964 136 - 22 064 323	188	21 914 136 - 22 114 323
2.	NAU 3284	chr. D_{11}	16 054 534 - 16 154 692	159	16 004 534 - 16 204 692
3.	NAU 2951	chr. A_{11}	6 595 544 - 6 695 709	166	6 545 544 - 6 745 709
		chr. D_{11}	6 024 909 - 6 125 074	166	5 974 909 - 6 175 074
4.	NAU 5212	chr. D_{11}	22 340 767 - 22 441 019	253	22 290 767 - 22 491 019
5.	BNL 3255	chr. A_{08}	112 193 008 - 112 293 216	209	112 143 008 - 112 343 216
6.	BNL 1604	chr. A_{07}	89 641 364 - 89 741 471	108	89 591 364 - 89 791 471
		chr. D_{07}	50 933 632 - 51 033 742	111	50 883 632 - 51 083 742
7.	BNL 1122	chr. A_{07}	45 473 898 - 45 574 059	162	45 423 898 - 45 624 059
			G. darwinii		
1.	NAU 3478	chr. D_{11}	216 690 110 - 21 769 197	188	216 640 110 - 21 819 197
2.	NAU 3284	chr. A_{11}	18 589 903 - 18 690 063	166	18 539 903 - 18 740 063
		chr. D_{11}	15 775 258 - 15 875 416	159	15 725 258 - 15 925 416
3.	NAU 2951	chr. A_{11}	6 427 204 - 6 527 369	166	6 377 204 - 6 577 369
		chr. D_{11}	5 911 615 - 6 011 780	166	5 861 615 - 6 061 780
4.	NAU 5212	chr. D_{11}	21 991 593 - 22 091 857	265	21 941 593 - 22 141 857
5.	BNL 3255	chr. A_{08}	110 842 283 - 110 942 489	207	110 792 283 - 110 992 489
6.	BNL 1604	chr. A_{07}	86 897 161 - 86 997 268	108	86 847 161 - 87 047 268

		chr. D_{07}	49 751 734 - 49 851 852	119	49 701 734 - 49 901 852
7.	BNL 1122	chr. A_{07}	43 072 317 - 43 172 478	162	43 022 317 - 43 222 478

Table S12. Information on putative genes and their transcripts identified in the marked regions using the AUGUSTUS web application

No.	SSR Marker	Number of Putative Genes	Number of Transcripts
1.	NAU3478	8	9
2.	NAU3284	11	11
3.	NAU2951	13	13
4.	NAU5212	10	10
5.	BNL3255	13	13
6.	BNL1604	9	10
7.	BNL1122	8	11
	Total:	72	77

Table S13. Putative genes identified in marker regions and the proteins they encode by means of BLAST analysis

№	SSR markers	Putative genes and/or encoded proteins
1.	NAU3478	1. *Proline-rich receptor-like protein kinase* (PERK4);<br>2. *G. hirsutum gypsy-like retroelement hrs7 integrase* gene;<br>3. *Putative caffeic acid methyltransferase* gene.
2.	NAU3284	1. *G. hirsutum gypsy-like retroelement hrs7 integrase* gene.
3.	NAU2951	1. *G. hirsutum glucose-6-phosphate/phosphate translocator 1* (GPT1);<br>2. *G. hirsutum AP-5 complex subunit zeta-1;*<br>3. *G. hirsutum serine carboxypeptidase-like proteins* 44 and 42;<br>4. *G. hirsutum protein disulfide-isomerase 5-3-like protein;*<br>5. *G. hirsutum early nodulin-like protein 3.*
4.	NAU5212	1. *Zinc finger BED domain-containing DAYSLEEPER-like protein;*<br>2. *G. hirsutum TRAF3-interacting protein 1-like protein.*
5.	BNL3255	1. *Putative FAD-binding oxidoreductase and protein disulfide-isomerase* genes;<br>2. *Putative caffeic acid methyltransferase* gene;<br>3. *Photosystem II protein* gene.

6.	BNL1604	1. *G. herbaceum retroelement hbc19 integrase* gene.
7.	BNL1122	1. *G. hirsutum zinc finger BED domain-containing DAYSLEEPER-like protein*.
